# Paramagnetic Relaxation Agents for Enhancing Temporal Resolution and Sensitivity in Multinuclear FlowNMR Spectroscopy

**DOI:** 10.1002/chem.202300215

**Published:** 2023-05-17

**Authors:** Alejandro Bara‐Estaún, Marie C. Harder, Catherine L. Lyall, John P. Lowe, Elizaveta Suturina, Ulrich Hintermair

**Affiliations:** ^1^ Department of Chemistry University of Bath Claverton Down Bath BA2 7AY United Kingdom; ^2^ Dynamic Reaction Monitoring Facility University of Bath, Claverton Down Bath BA2 7AY United Kingdom; ^3^ Institute for Sustainability University of Bath Bath BA2 7AY United Kingdom

**Keywords:** chromium, flowNMR, multinuclear NMR spectroscopy, spin-lattice relaxation time and paramagnetic relaxation agents

## Abstract

Sensitivity in FlowNMR spectroscopy for reaction monitoring often suffers from low levels of pre‐magnetisation due to limited residence times of the sample in the magnetic field. While this *in‐flow* effect is tolerable for high sensitivity nuclei such as ^1^H and ^19^F, it significantly reduces the signal‐to‐noise ratio in ^31^P and ^13^C spectra, making FlowNMR impractical for low sensititvity nuclei at low concentrations. Paramagnetic relaxation agents (PRAs), which enhance polarisation and spin‐lattice relaxation, could eliminate the adverse *in‐flow* effect and improve the signal‐to‐noise ratio. Herein, [Co(acac)_3_], [Mn(acac)_3_], [Fe(acac)_3_]_,_ [Cr(acac)_3_]_,_ [Ni(acac)_2_]_3,_ [Gd(tmhd)_3_] and [Cr(tmhd)_3_] are investigated for their effectiveness in improving signal intensity per unit time in FlowNMR applications under the additional constraint of chemical inertness towards catalytically active transition metal complexes. High‐spin Cr(III) acetylacetonates emerged as the most effective compounds, successfully reducing ^31^P T_1_ values four‐ to five‐fold at PRA concentrations as low as 10 mM without causing adverse line broadening. Whereas [Cr(acac)_3_] showed signs of chemical reactivity with a mixture of triphenylphosphine, triphenylphosphine oxide and triphenylphosphate over the course of several hours at 80° C, the bulkier [Cr(tmhd)_3_] was stable and equally effective as a PRA under these conditions. Compatibility with a range of representative transition metal complexes often used in homogeneous catalysis has been investigated, and application of [Cr(tmhd)_3_] in significantly improving ^1^H and ^31^P{^1^H} FlowNMR data quality in a Rh‐catalysed hydroformylation reaction has been demonstrated. With the PRA added, ^13^C relaxation times were reduced more than six‐fold, allowing quantitative reaction monitoring of substrate consumption and product formation by ^13^C{^1^H} FlowNMR spectroscopy at natural abundance.

## Graphical abstract

**With the right amount of an easily available chromium(III) complex** bearing a bulky acetylacetonato ligand to keep it chemically inert, NMR spectra acquired in continuous flow give much better data due to significantly reduced polarisation and relaxation times without undue peak broadening or loss of 2D methods.

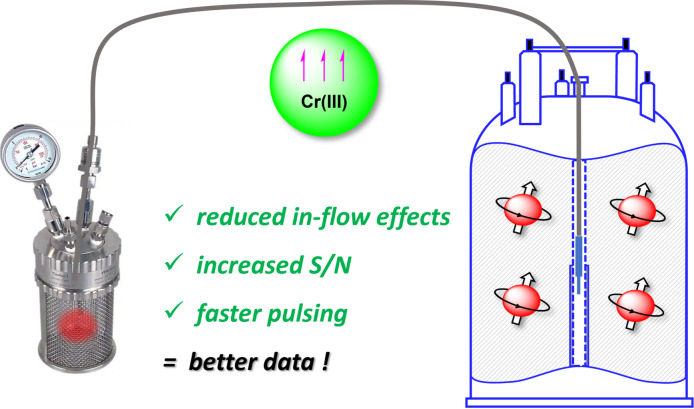

## Introduction

Nuclear magnetic resonance spectroscopy (NMR) is one of the most widely used and most powerful analytical techniques in synthetic chemistry due to its wide applicability, its inherent quantitative nature and the level of structural information provided.[^1^, ^2^, ^3^] The main drawback of NMR is its low sensitivity compared to other analytical methods; the level of detection for NMR is ∼5×10^−9^ mol whereas mass spectrometry has achieved 10^−19^ mol.^[4]^ Despite this, NMR is routinely employed as an information‐rich ex situ characterisation technique, and state‐of‐the‐art NMR methods allow for monitoring reactions in situ or online.^5^ FlowNMR techniques, where an aliquot from an external reaction vessel is pumped through the spectrometer, are particularly useful for investigating reactive systems in their native environments under realistic conditions.^[6]^ Although being investigated since the 1960s[^7^, ^8^, ^9^] FlowNMR spectroscopy has recently flourished as a popular reaction monitoring technique,[^10^, ^11^, ^12^, ^13^] partly due to the commercialisation of suitable hardware. The use of high resolution, multinuclear NMR techniques has been shown to be a powerful tool for mechanistic investigations in homogeneous catalysis^[14]^ including photoredox, polymerization, transfer hydrogenation and hydroformylation reactions, along with in vivo and metabolomics studies.[^14^, ^15^, ^16^, ^17^, ^18^, ^19^, ^20^, ^21^, ^22^, ^23^, ^24^, ^25^]

In addition to the engineering considerations of designing an effective FlowNMR setup,^[26]^ flow effects on the NMR signal acquisition need to be considered to generate accurate results.^[14]^ The so‐called *in‐flow* effect, caused by partial pre‐magnetisation of nuclei entering the NMR detection region, reduces signal intensities if the residence time of the spins within the magnet is shorter than five times their spin‐lattice relaxation time (T_1_) since the thermal spin polarisation cannot be reached.^[27]^ This effect may be accounted for with flow correction factors for integrals of interest, and is thus not much of a problem for nuclei with high gyromagnetic ratio and high abundance (e. g. ^1^H or ^19^F). However, the *in‐flow* effect lowers FlowNMR sensitivity for heteronuclei with low gyromagnetic ratio and/or low abundance such as ^31^P and ^13^C to a degree where mechanistic investigations on fleeting intermediates at low concentration become challenging or even impossible.^[14]^ This limitation is exacerbated by the requirement for time‐resolved measurements in a FlowNMR experiment following a batch reaction proceeding at its intrinsic rate under the conditions applied. Hence in addition to the signal‐reducing *in‐flow* effect, maximising signal intensity per unit time is a fundamental challenge for kinetic FlowNMR investigations. The so‐called *out‐flow* effect provides some temporal enhancement of multi‐scan acquisitions in flow compared to static conditions due to the continuous displacement of saturated spins with fresh material (thereby shortening the apparent spin‐lattice relaxation time T_1_* and allowing faster pulsing).^[14]^ However, the improvement provided by the *out‐flow* effect for heteronuclei with long T_1_ times, low gyromagnetic ratio and low isotopic abundance cannot compensate for the detrimental loss of spin polarisation due to the *in‐flow* effect.

Stable open‐shell molecules based on organic radicals or paramagnetic transition metal complexes possess a magnetic moment approximately 250 times that of ^1^H nuclei.^[27]^ When added to a solution NMR sample at low concentrations these compounds cause paramagnetic relaxation enhancement that reduces the nuclear spin‐lattice relaxation time of diamagnetic analytes,^[7]^ an effect that is widely used to enhance contrast in magnetic resonance imaging (MRI).[^28^, ^29^, ^30^, ^31^, ^32^] The dipole interactions between the unpaired electron(s) of the paramagnetic relaxation agent (PRA) and any surrounding nuclear spins are strongly distance‐dependant as the paramagnetic relaxation effect scales with *r* ^−6^.^[33]^ Since both longitudinal relaxation times (T_1_) and transverse relaxation times (T_2_) are reduced, NMR signals of nuclei very close to the paramagnetic centre may become so broad that they are no longer observable in the NMR spectrum.^[34]^

Paramagnetic transition metal complexes have previously been used to enhance nuclear magnetic relaxation for improved kinetic NMR measurements. In 2003, Fischer et al. reported the use of surface‐grafted gadolinium(III) complexes as immobilised PRAs to reduce longitudinal relaxation times and increase sensitivity of ^13^C{^1^H} FlowNMR.^[35]^ In 2009 Denmark et al. used [Cr(tmhd)_3_] to shorten the T_1_ of ^31^P and ^29^Si nuclei to allow for faster and more accurate acquisition in rapid injection stopped‐flow NMR to investigate Lewis base‐catalysed aldol addition reactions.^[36]^ More recently in 2021 Kircher et al. studied the signal enhancement by Overhauser dynamic nuclear polarization (ODNP) based on the polarization transfer of paramagnetic species to enable fast‐flow benchtop NMR spectroscopy.^[37]^ Here we investigate the utility of some commonly used PRAs based on coordinatively saturated transition metal complexes in providing a suitable level of magnetic coupling that reduces the T_1_ times of ^1^H, ^31^P and ^13^C for continuous FlowNMR applications in reaction monitoring of homogeneous catalysis. We show how *in‐flow* effects can be reduced and signal intensities per unit time increased without causing adverse line broadening effects or affecting the ability to perform multi‐dimensional correlation experiments. Aspects of solubility, spectral resolution, chemical inertness, and compatibility with reactive systems have also been considered.

## Results and Discussion

### PRA selection

**Figure 1:**
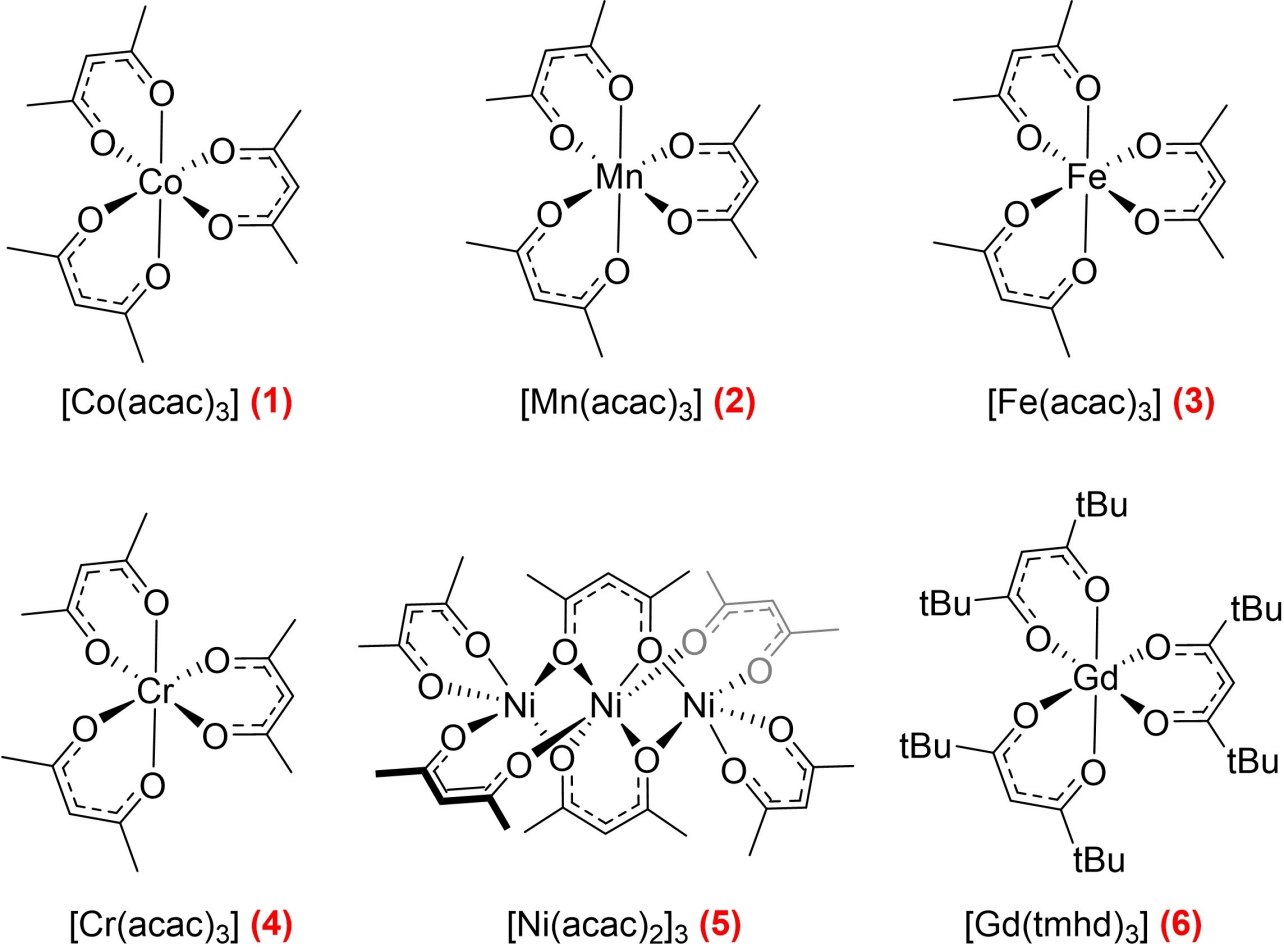
Structures of the metal complexes investigated as chemically inert paramagnetic relaxation agents in heteronuclear FlowNMR spectroscopy.

Six transition metal complexes (Figure 1) were initially selected as potential paramagnetic relaxation agents from the literature based on their electronic configuration, reactivity, and availability or ease of synthesis.[^38^, ^39^, ^40^, ^41^, ^42^, ^43^, ^44^, ^45^, ^46^, ^47^] Although all of these compounds have previously been used as PRAs or MRI contrast agents, here we have evaluated the reduction of the *in‐flow* effect in multinuclear high‐resolution FlowNMR spectroscopy in reactive mixtures containing ligands and metal complexes often used in homogeneous catalysis. In this context, we have also evaluated their chemical inertness, solubility in organic solvents, and spectroscopic interference.[^48^, ^49^]

### Phosphorous model compounds

Triphenylphosphine (TPP), triphenylphosphine oxide (TPPO) and triphenylphosphate (TPOP) were selected as model compounds to test for chemical compatibility with homogeneous catalytic systems. TPP is a widely used ligand in homogeneous transition metal catalysis with good σ‐donor and π‐acceptor properties, its oxide TPPO a good ligand for harder, more ionic Lewis acids, and the phosphate TPOP is a useful internal standard for quantitative ^31^P NMR spectroscopy.^[51]^ Their ^31^P NMR spectra and longitudinal relaxation times were initially measured under static conditions in normal sample tubes as all three compounds have easily distinguishable, sharp singlet peaks in the ^31^P{^1^H} NMR spectrum at −5.2, 24.3 and −17.4 ppm, respectively (Figure S1). Control experiments showed the stability of this mixture with no spectral changes over extended periods of time, increased temperature and exposure to air (Figure S2 and Table S5).

**Figure 2:**
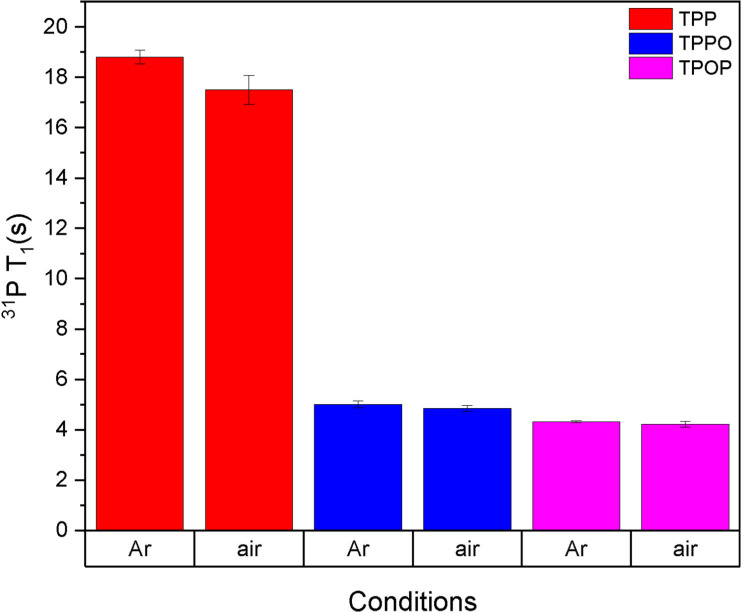
^31^P T_1_ values of TPP, TPPO and TPOP as measured by recovery inversion experiments at 298 K on a sample containing 10 mM of TPP, TPPO and TPOP in 0.5 mL of dry toluene.

Spin‐lattice relaxation time constants (T_1_) are known to be affected by temperature, concentration, solvent viscosity and polarity, as well as the solution concentration of O_2_.^[52]^ Quantification of the T_1_ values of TPP, TPPO and TPOP via inversion recovery experiments^[53]^ under the conditions detailed in the methodology section yielded values consistent with literature^[54]^ that span a range of relaxation times (Figure 2 and Table S6) that lead to sizable *in‐flow* effects due to incomplete pre‐magnetisation in FlowNMR with typical flow rates of 2–4 mL/min.

As shown in Figure 2, the T_1_ values for the three species were relatively large, TPPO and TPOP being similar but TPP relaxing much slower. For a quantitative spectrum, the recycle time of the experiment must be at least 5 times the longest T_1_ of the nuclei of interest,^[55]^ meaning that the TPP signals would be affected the most in this example. The ∼5 % reduction of T_1_ observed in air compared to anaerobic conditions may be ascribed to the weakly paramagnetic nature of O_2_.^[56]^ All T_1_ values decreased after the sample was heated up at 353 K (Table S7), an effect mainly due to the reduction of solvent viscosity and a higher rate of tumbling.^[57]^

### PRA compatibility testing

**Figure 3:**
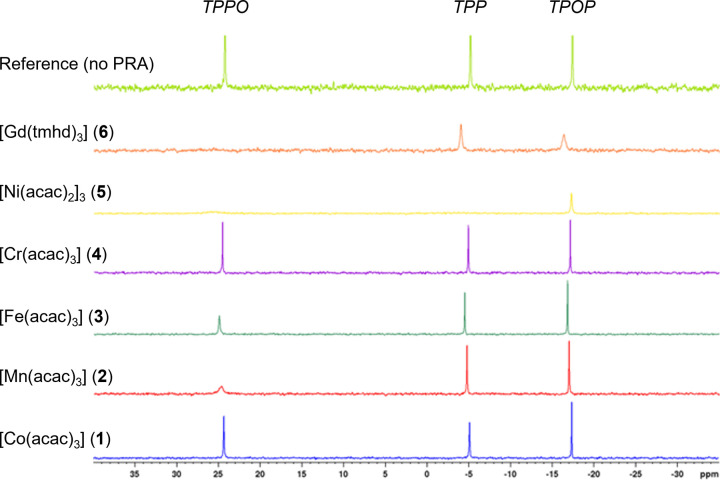
^31^P{^1^H} NMR spectra of samples containing 10 mM of TPP, TPPO, TPOP plus 10 mM of PRA as specified in 0.5 mL of dry toluene recorded at 298 K under Argon (NS=16, D_1_=2 sec, AQ=0.81 sec, O_1_P=0 ppm, SW=200 ppm).

To assess the chemical compatibility of the PRA candidates **1**–**6** with the mixture of TPP, TPPO and TPOP they were combined in toluene at equimolar quantities and the ^31^P{^1^H} NMR spectra monitored (Figure 3). Most of the samples showed significant changes in chemical shift or signal intensities due to interaction with the paramagnetic transition metal centre immediately after mixing. Complexes **2**, **3**, **5** and **6** quickly reacted with TPPO, causing its disappearance from the ^31^P{^1^H} NMR spectra. Whereas this reactivity may have been expected from the large lanthanide in **6** and the labile trimer of **5**, the observation of ligand exchange with the octahedral Mn complex **2** and the octahedral Fe complex **3** is perhaps less obvious. Complexes **5** and **6** also reacted with TPP and TPOP, causing significant peak broadening and reduction of signal intensity. The Co complex **1** appeared to cause only few changes to the mixture, but closer inspection of the NMR spectra showed diminishing TPP integrals over time. The Cr complex **4** was found to be the only candidate tested that caused no discernible changes to the appearance of the ^31^P{^1^H} NMR spectra of the mixture under the conditions applied. More details, including a comparison of peak intensities, chemical shifts and signal linewidths of all samples can be found in the Supporting Information (Table S8).

### [Cr(acac)_3_] as outer‐sphere PRA

Two types of paramagnetic relaxation mechanisms can be distinguished: intramolecular and intermolecular. Intramolecular or inner‐sphere relaxation occurs when the interacting spins, nuclear (*I*) and electron (*S*), are part of the same molecule to give strong magnetic coupling through orbital overlap. An example of this type is solvent molecules reversibly binding to a paramagnetic transition metal centre, as for example used in MRI applications, which leads to severe peak broadening or even complete disappearance of the NMR signals due to very fast relaxation. Intermolecular or outer‐sphere relaxation caused by dipole‐dipole interactions between discrete molecules modulated by translational diffusion leads to lower levels of relaxation enhancement that typically preserves the NMR signals.[^29^, ^58^] The difference between inner‐sphere and outer‐sphere relaxation enhancement depends on the affinity of the analyte of interest to the paramagnetic transition metal centre and the relative coordination strengths of the ligands used.[^41^, ^59^] Inner‐sphere relaxation is likely the reason behind the partial signal disappearance observed with some complexes (Figure 3), which is often seen when using coordinatively unsaturated or labile PRAs with analytes of varying donor power.^[33]^

If both analyte and PRA are inert and their diffusion in the medium with viscosity η can be modelled as that of rigid spheres with radii $a_{1}$ and $a_{2}$ , the outer‐sphere relaxation enhancement can then be described by Abragam's Equation 1

(1)

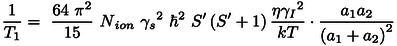




This expression captures the effect of temperature (T ), solvent viscosity (η ) and PRA concentration ($N_{ion\;}$ ) on the longitudinal relaxation time constants ($T_{1}$ ) of surrounding analytes caused by a PRA with gyromagnetic ratio ($\gamma_{s}$ ) and spin (*S’*).^[60]^

#### Effect of concentration

**Figure 4:**
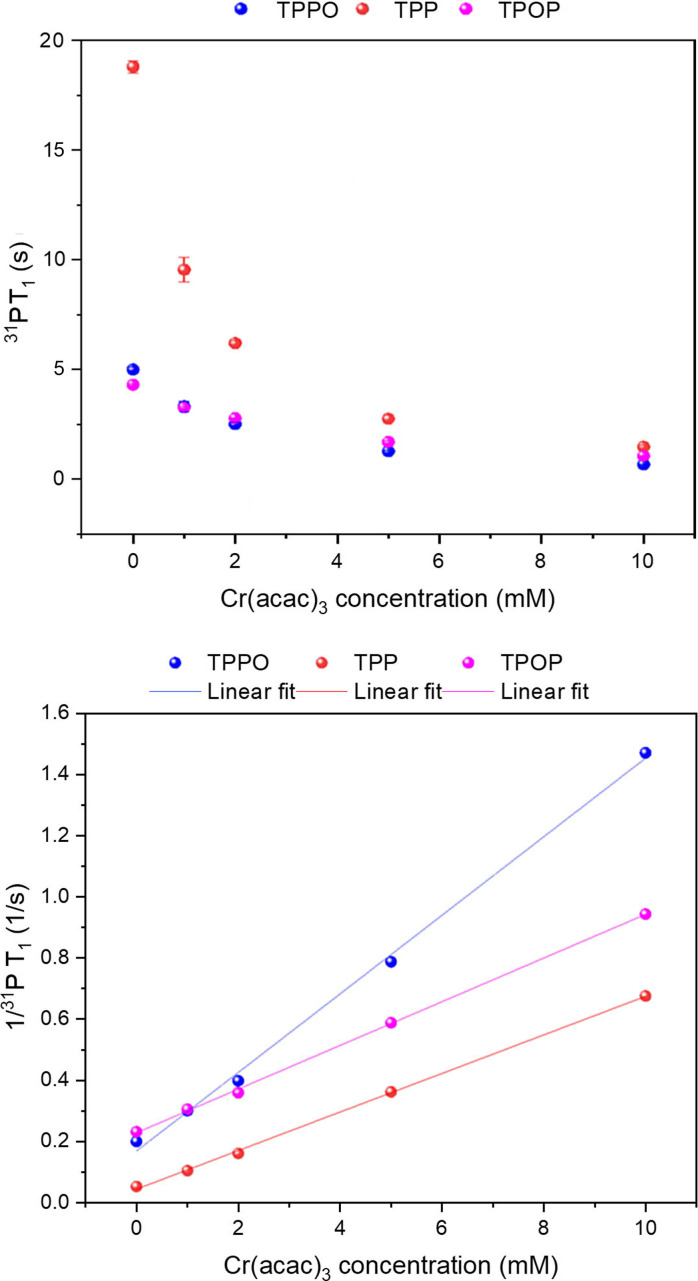
^31^P T_1_ (top) and 1/T_1_ (bottom) values of TPP, TPPO and TPOP as measured by inversion recovery experiments at 298 K in samples containing 10 mM of each analyte with 0, 1, 2, 5 and 10 mM of [Cr(acac)_3_] in dry toluene under Argon (for fit parameters see Figure S3)

To optimise the level of relaxation enhancement for alleviating sensitivity limitations from *in‐flow* effects and increase temporal resolution without negatively impacting spectral quality by adverse line broadening, a suitable concentration regime needs to be defined. Assessing spectral quality and spin‐lattice relaxation times via ^1^H, ^31^P{^1^H} and ^31^P{^1^H} inversion recovery experiments at room temperature under argon on samples with varying concentrations of [Cr(acac)_3_] (**4**) at constant TPP, TPPO, and TPOP concentration allowed the evaluation of chemical reactivity and relaxation enhancement at different loadings (Figure 4 & Table S9). As can be seen from the data, even at a 10 % loading **4** was effective at halving the T_1_ of TPP from 18.8±0.3 s down to 9.6±0.6 s, effectively doubling the temporal resolution of a quantitative ^31^P{^1^H} NMR experiment. Smaller reductions were experienced by the more quickly relaxing analytes TPPO and TPOP, but all three observed T_1_ values converged to a common level of 1.5–0.7 s at an equimolar loading of 10 mM **4**, corresponding to significant spin‐lattice relaxation enhancements of 75–92 %. Plotting 1/T_1_ over PRA concentration showed the linear relationship expected from Equation (1), with slopes of 0.13 s^−1^ (TPPO), 0.07 s^−1^ (TPOP) and 0.06 s^−1^ (TPP) (Figure S3) that reflected the difference in hydrodynamic size of the molecules in solution. The values obtained were in line with those reported by Carr et al. when using the same analytes with Fe(acac)_3_.^[33]^ Repeating the analysis in air instead of argon gave similar results (Table S10).

Importantly, the PRA minimised the range of T_1_ values across the mixture, which is particularly useful for reaction monitoring by heteronuclear NMR spectroscopy, as acquisition parameters may be adjusted more tightly for maximum S/N per unit time while remaining quantitative across all signals. With a 4 mL/min sample flow through a 11.7 T magnet in 0.7 mm I.D. tubing^[26]^ the addition of 10 mM of [Cr(acac)_3_] would increase the level of magnetic polarisation of TPP from 19 % to 93 % (see Supporting Information section 5), corresponding to an almost five‐fold increase in ^31^P FlowNMR sensitivity per unit time.

Although the aim is to enhance spin‐lattice relaxation and thereby decrease the overall measurement time, the signal‐to‐noise ratio (S/N) of the NMR signals obtained must not be neglected. Enhancement of spin‐lattice relaxation goes hand in hand with enhancement of spin‐spin relaxation^[61]^ that might lead to adverse line broadening and thus lower S/N that decreases the accuracy of peak integration. Fortunately, the ^31^P{^1^H} NMR peak widths of TPP, TPPO and TPOP showed no adverse increases up to 10 mM **4** under the conditions applied (Figure S4).

#### Effect of temperature

Online reaction monitoring typically requires the use of specific reaction temperatures for the analysis, and like NMR signal intensities and T_1_ values, the effectiveness of PRAs is also temperature‐dependent through changes in solvent viscosity and magnetic moment of the PRA (Eq. (1)).[^59^, ^62^] Furthermore, for reaction monitoring applications it is important to ensure the chemical inertness of the PRA under the conditions applied, as elevated temperatures may lead to unwanted reactivity such as ligand exchange that could cause the loss of relevant NMR signals from the spectrum (as seen with some of the less suitable PRAs, Figure 3). Using an equimolar ratio of **4** to each analyte at 10 mM, variable temperature (VT) NMR experiments were thus carried out from 273 to 353 K in different step sizes (Tables S11 & S12). Whereas over the course of a short VT experiment (4 h) using 20 K steps the T_1_ values of all three analytes increased linearly when going to higher temperatures (Figure S5), with 5 K steps and thus longer total heating times (12 h) non‐linear behaviour was observed for TPP and TPPO (Figure S6).

This apparent reduction of T_1_ in TPP and TPPO at higher temperatures can be ascribed to a change from outer‐sphere to inner‐sphere relaxation due partial coordination of the ligands to the paramagnetic Cr^III^ centre at elevated temperatures, consistent with increased peak widths and decreased integral values for TPP and TPPO in these spectra.^[29]^ Thus, while [Cr(acac)_3_] is a suitable outer‐sphere PRA for reaction monitoring applications at room temperature it seems to react with even moderately basic ligands at higher temperatures.

### [Cr(tmhd)_3_] as inert outer‐sphere PRA

**Figure 5:**
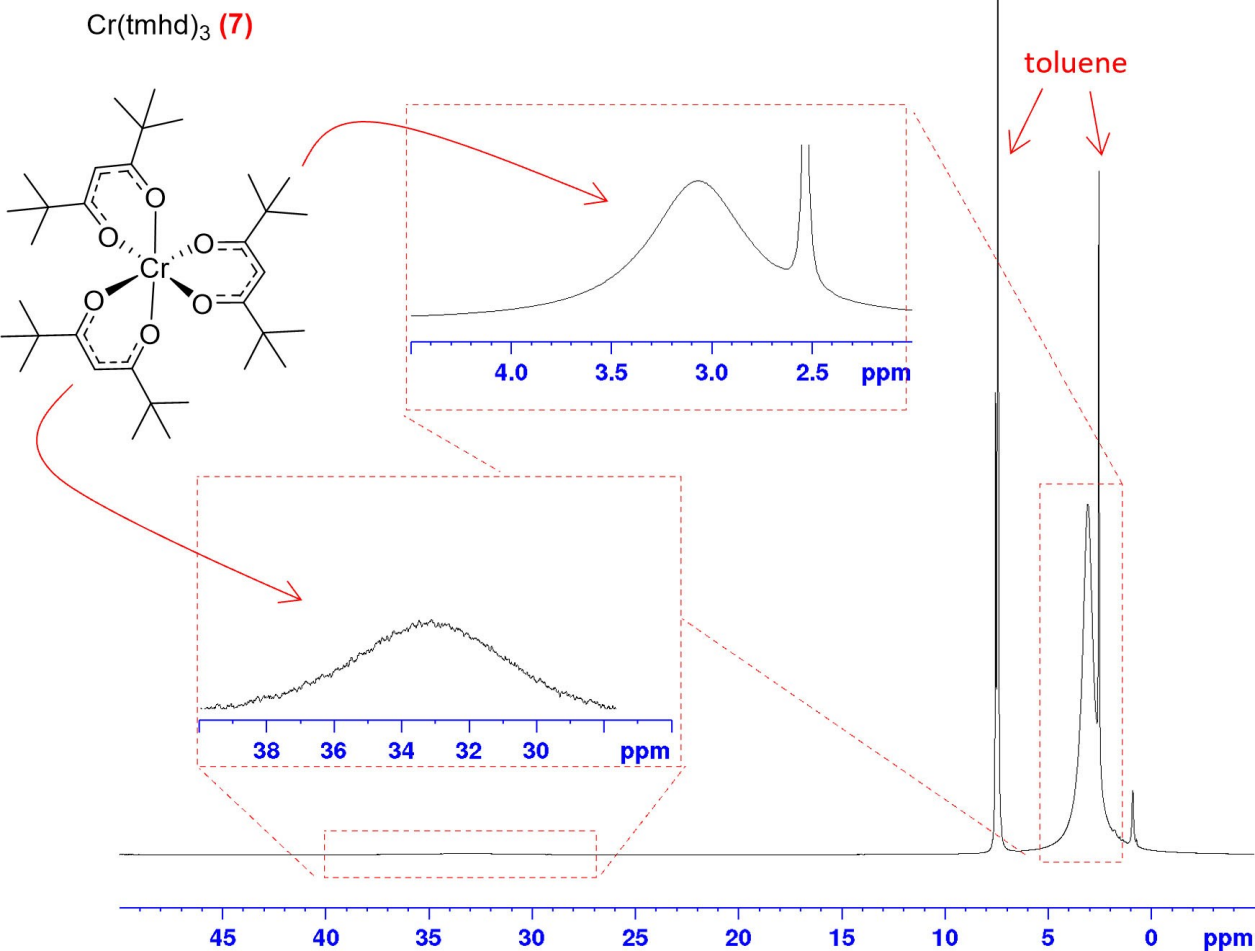
Molecular structure and ^1^H NMR spectrum of [Cr(tmhd)_3_] at 25 mM in deuterated toluene (NS=64, D_1_= 1 sec, AQ=0.64 sec, O_1_P=6 ppm, SW=600 ppm).

We thus turned to [Cr(tmhd)_3_] (**7**) as a bulkier version of [Cr(acac)_3_] that has also previously been used as a PRA,^[39]^ including in reaction monitoring by rapid injection ^31^P{^1^H} NMR spectroscopy,^[36]^ in the hope that the increased steric bulk of the ^t^Bu groups would prevent ligand association to the octahedral 15‐electron Cr^III^ centre. The paramagnetic ^1^H NMR spectrum of **7** showed one reasonably sharp signal for the 54 butyl protons at 2.3 ppm (FWHM 333.5 Hz) and one very broad resonance for the three methine protons around 33 ppm due to their closer proximity to the Cr centre (Figure 5). No ^13^C NMR signals were observable at 25 mM by ^13^C{^1^H} and ^1^H‐^13^C HMBC spectroscopy *(NS=16, DS=16, D*_*1=*_ *1.33 sec, AQ=0.4 sec, O_1_P=(5.5 ppm [^1^H], 100 ppm [^13^C]), SW=(20 ppm [^1^H], 400 ppm [^13^C]))*.

The UV‐vis spectrum of **7** in toluene showed strong UV absorption below 430 nm and a moderate intensity band in the visible region at 570 nm (Figure S7), and it was found to be readily soluble in toluene, chloroform, THF, acetone, acetonitrile and methanol at 10 mM at room temperature.^[63]^

#### Chemical stability

To assess the chemical reactivity of **7**, samples of the three model analytes TPP, TPPO and TPOP at 10 mM in toluene were mixed with an equimolar amount of [Cr(tmhd)_3_] and heated to 353 K. Even after three weeks at 80 °C no changes in peak positions, line widths and integral areas were noticeable (Table S14 & S15), showing the bulky tmhd ligand in **7** to confer enhanced stability to the Cr^III^ centre compared to the simple acac ligand in **4**.

#### Effectiveness of relaxation

Quantifying the magnetic relaxation efficiency of **7** on TPP, TPPO and TPOP by ^31^P inversion recovery experiments showed very similar levels of T_1_ reduction as those found with **4**: at 10 mM concentration in toluene at room temperature all ^31^P T_1_ values decreased by up to 91 % down to a range of 1.6–1.2 s (Figure S8 & Table S13), and the same concentration dependency was observed as for [Cr(acac)_3_] (Figure S9 & Table S16). The marginally lower levels of relaxation afforded by **7** compared to **4** is probably due to the larger size of the bulky tmhd ligand that slows its diffusion and increases the distance between analytes and the paramagnetic Cr^III^ centre. As with **4**, peak widths were not affected in the range of concentrations investigated.

#### Effect of temperature

**Figure 6:**
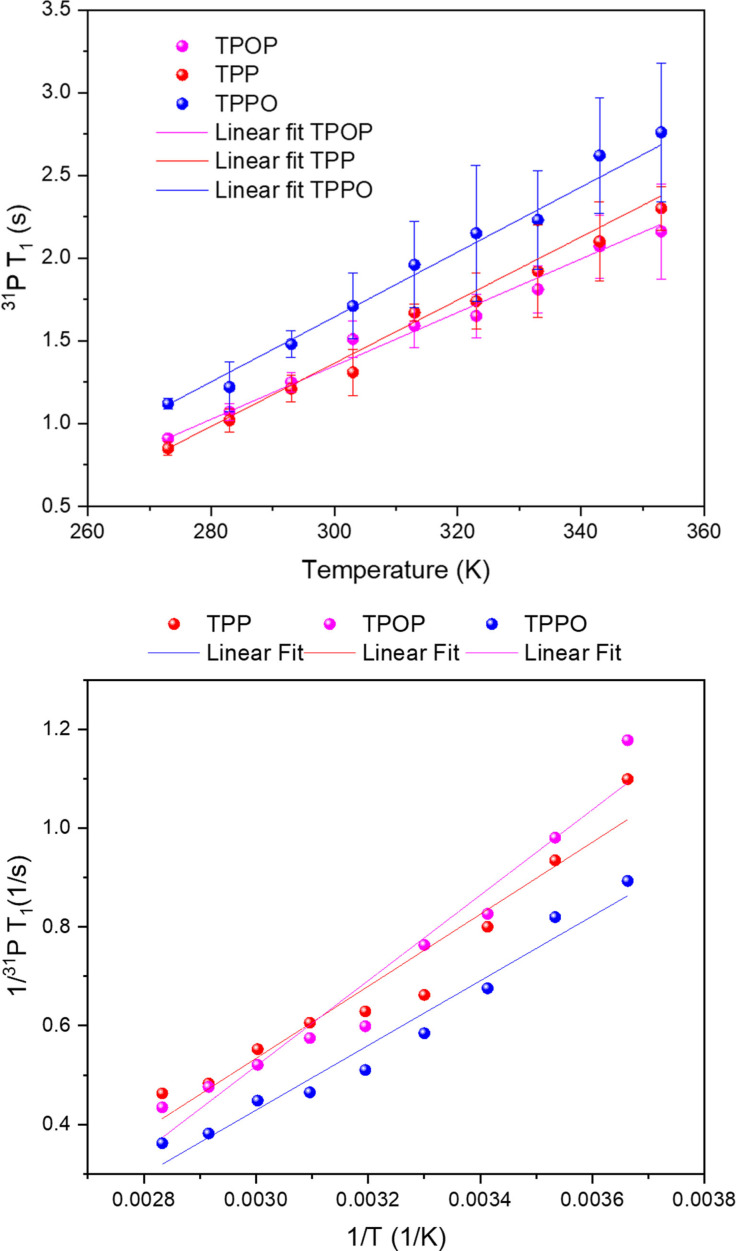
^31^P T_1_ (top) and 1/T_1_ (bottom) of TPP, TPPO and TPOP as measured by inversion recovery experiments at different temperatures in samples containing 10 mM of each analyte with 10 mM of [Cr(tmhd)_3_] in dry toluene under Argon (for fit parameters see Figure S11)

With a stable and effective PRA in hand we revisited the effect of temperature on the outer‐sphere relaxation efficacy of **7**. Measuring ^31^P T_1_ values of TPP, TPPO and TPOP with the inert [Cr(tmhd)_3_] at different temperatures produced a linear increase in all spin‐lattice relaxation times, or decrease in relaxation efficiency, when increasing the sample temperature (Figure 6 & Table S17). In line with the stability measurements (2.3.1), returning to any sample temperature reproduced the T_1_ values initially found, showing any changes to be fully reversible temperature effects. Relative changes in T_1_ times were similar for all three analytes, with gradients in the order of 16–20 ms/K. Again, this is beneficial for reaction monitoring applications where a narrow distribution of T_1_ values across all species is advantageous. The absolute levels of relaxation afforded by 10 mM **7** at 80 °C are 65 to 89 %. If higher levels of relaxation are required at elevated temperatures higher concentrations of **7** may be used (see Figure S9).

The collective behaviour of **7** at different concentrations and temperatures confirms the intermolecular, outer‐sphere relaxation of other species in solution (Eq. (1)).

#### Effects on ^1^H, ^13^C{^1^H} and 2D NMR experiments

**Figure 7:**
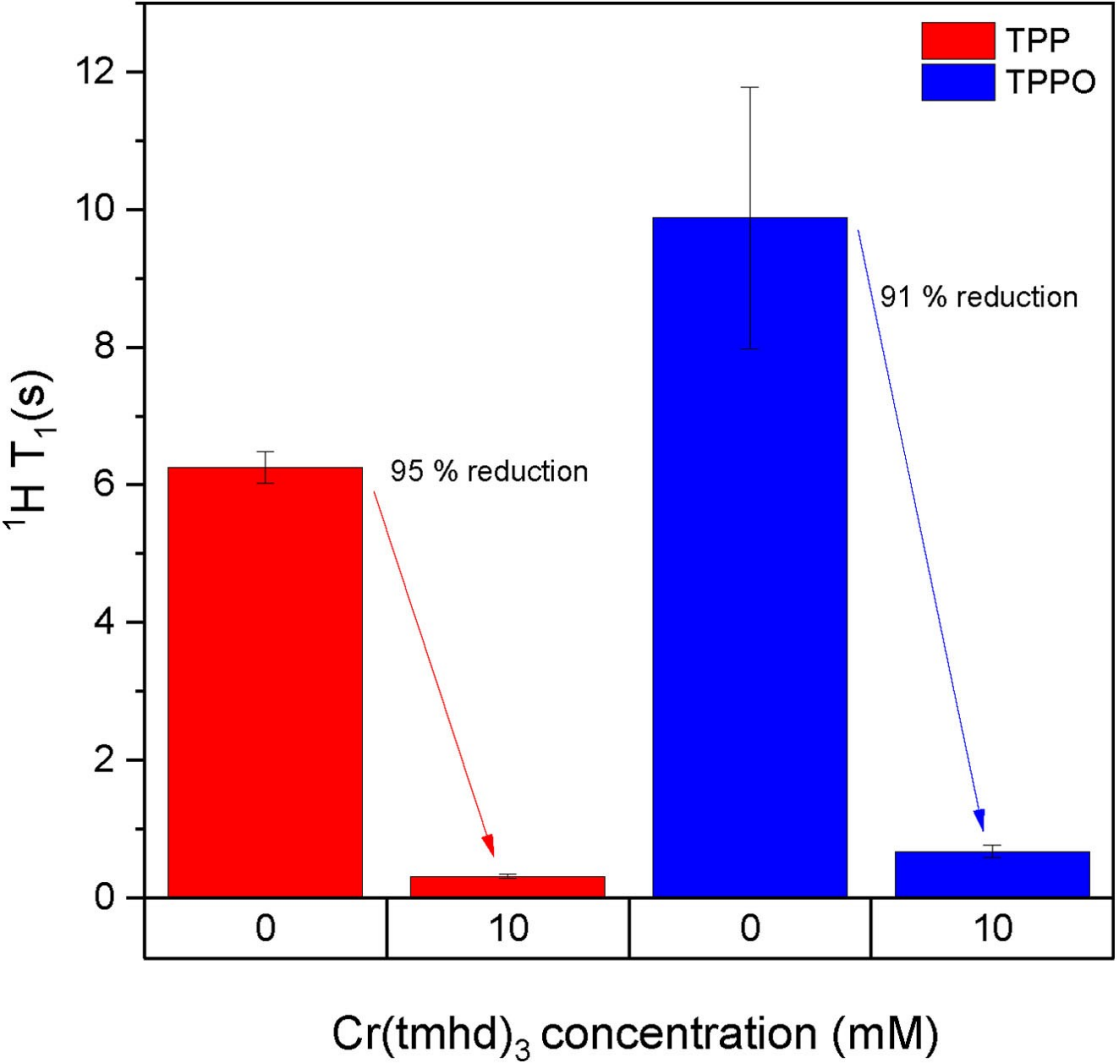
^1^H T_1_ values of TPP and TPPO as measured by inversion recovery experiments at 298 K in individual samples containing 10 mM analyte with and without [Cr(tmhd)_3_] in dry toluene under argon.

Quantitative ^1^H NMR experiments are often used to follow conversions and selectivities of reactions investigated by FlowNMR, and although generally much more sensitive than ^31^P NMR there is still room for improvement in reducing *in‐flow* effects and narrowing the distribution of T_1_ values across multiple peaks of interest. When a non‐specific, outer‐sphere PRA such as **7** is added to a reaction mixture to accelerate magnetisation build‐up and relaxation of ^31^P other nuclei will inevitably be affected too, so we investigated the effects of **7** on the ^1^H and ^13^C{^1^H} NMR signals of TPP and TPPO at 10 mM concentration (Figure 7 & Table S18).

The aromatic protons of TPP and TPPO (overlapping multiplets of all three positions) have relatively long longitudinal relaxation times of 6–10 seconds which were reduced by over 90 % to less than 1 second in the presence of one equivalent of **7**. Pleasingly, this reduction did not entail any noticeable changes in chemical shift position, multiplicity or peak linewidth of the signals, indicating that the transverse T_2_ relaxation times were not adversely affected by **7** under the conditions applied (Table S19).

**Figure 8:**
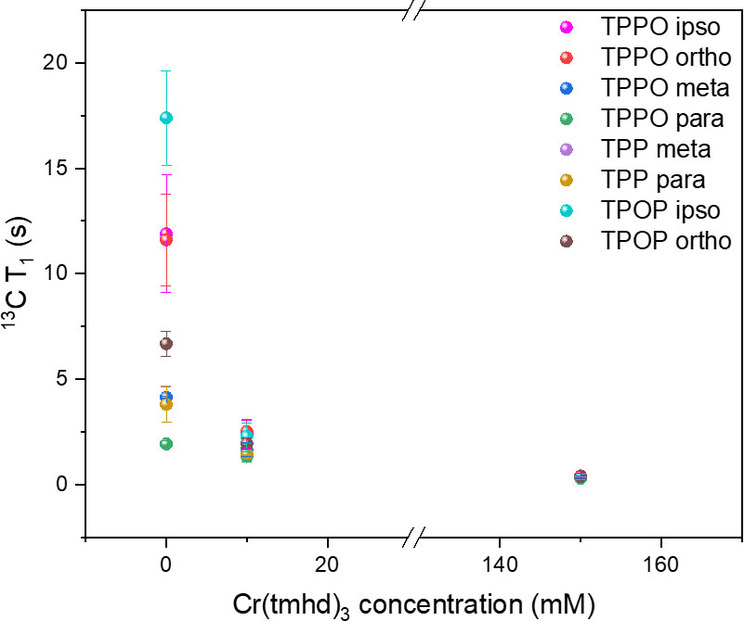
^13^C T_1_ values of TPP, TPPO and TPOP as measured by recovery inversion experiments at 298 K in individual samples of TPP, TPPO and TPOP at 150 mM with various amounts of [Cr(tmhd)_3_]in dry toluene under argon (inverse plot with linear fits in Figure S11).

^13^C is a potentially very useful but challenging nucleus for FlowNMR spectroscopy because of its low abundance and long spin‐lattice relaxation times.^[49]^ In order to obtain useful ^13^C NMR spectra of dilute or transient species a high number of scans must be applied to collect practically useful levels of signal intensity. In addition, long recycle times must be used for accurate quantitation. These requirements often clash with the demands of temporal resolution in reaction monitoring applications, resulting in very limited use of ^13^C FlowNMR at natural abundance. While PRAs cannot address the issue of low abundance, they are useful in reducing the particularly long T_1_ times of ^13^C as in the case of ^31^P. ^13^C{^1^H} inversion recovery experiments on individual samples of TPPO, TPP and TPOP at 150 mM with either 10 or 150 mM of [Cr(tmhd)_3_] showed significant, concentration‐dependent reductions of T_1_ values across the various aromatic carbon signals (Figure 8 & Table S20–22).

**7** was effective in reducing ^13^C spin‐lattice relaxation times by up to 95 % to afford a narrow distribution of short T_1_ times around 0.3 seconds at 150 mM, making ^13^C{^1^H} NMR spectroscopy at natural abundance fast and quantitative.^[36]^ Even at 10 mM the PRA was effective to afford reduced levels of T_1_ times of 1–3 s across all aromatic signals (including quaternary carbons) in the analytes present at 150 mM.

With the useful levels of paramagnetic relaxation enhancement afforded by **7** we wondered about its impact on the ability to carry out heteronuclear 2D correlation experiments. The cross‐peaks observed in ^1^H‐^31^P and ^1^H‐^13^C HMBC spectra of TPP, TPPO and TPOP were enhanced in intensity by 30 % through the addition of 10 mM [Cr(tmhd)_3_] without any observable changes in their chemical shift positions or peak widths (Table S23). This observation not only preserves the option of structure elucidation via correlation spectroscopy in the presence of **7** but opens the door to monitor reactions with fast and quantitative 2D experiments.[^47^, ^64^, ^65^]

#### Compatibility with transition metal complexes

**Figure 9:**
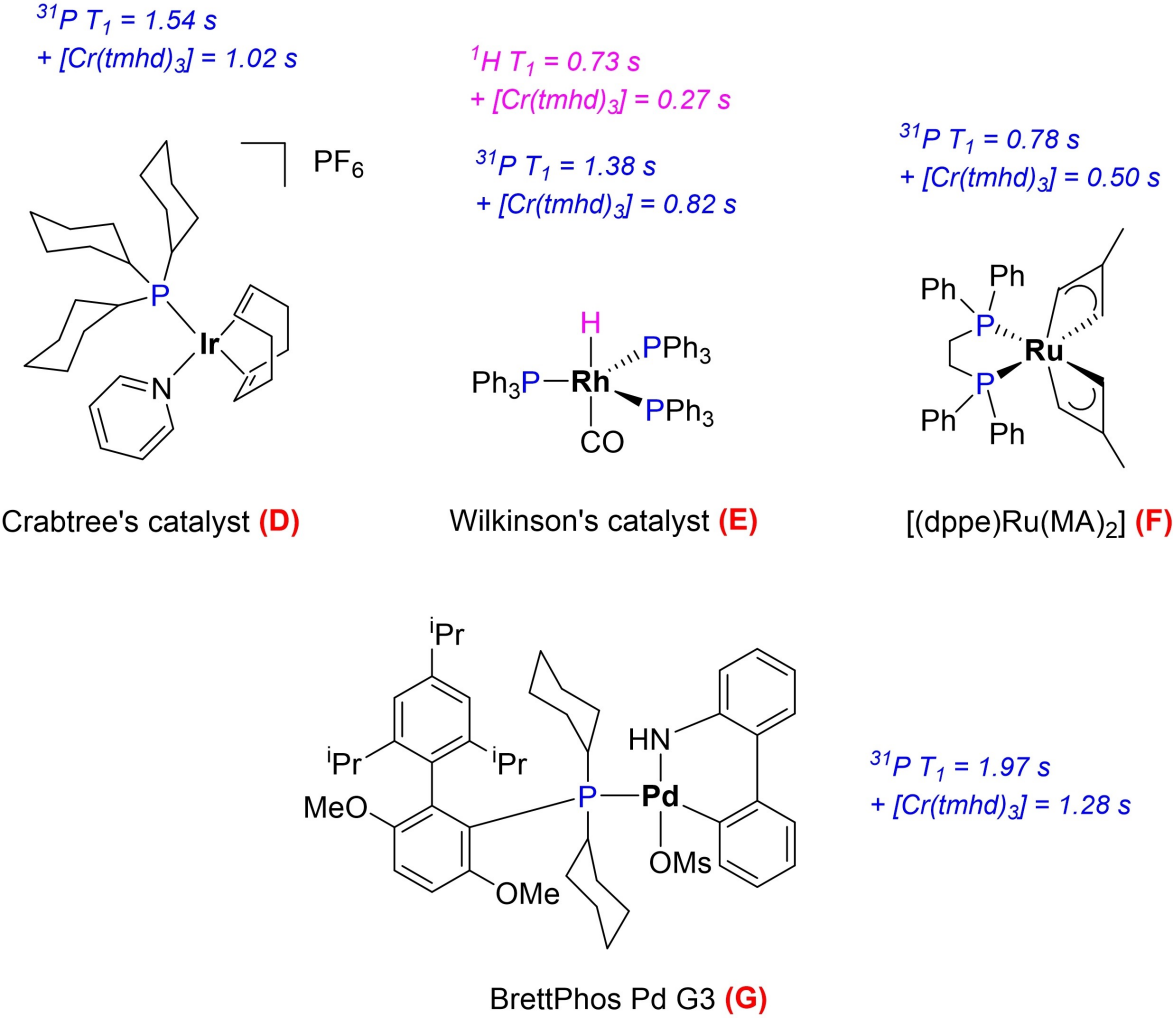
Transition metal complexes evaluated for compatibility with [Cr(tmhd)_3_]. ^31^P T_1_ values of **D**, **E**, **F** and **G** and ^1^H T_1_ values of **E** as measured by inversion recovery experiments at 298 K in individual samples containing 10 mM complex with and without 10 mM of [Cr(tmhd)_3_] in dry toluene under argon.

For use in reaction monitoring, an effective outer‐sphere PRA should also be inert towards reactive transition metal complexes often used in homogeneous catalysis. We thus tested the behaviour of **7** with four representative examples of late d‐block complexes frequently employed as precursors for catalytic hydrogenation (**D**), hydroformylation (**E**), rearrangement (**F**) and cross‐coupling (**G**) reactions (Figure 9). Although the reductions in T_1_ times of the various ^31^P and representative ^1^H signals were more modest compared to the levels seen with the free P^III^ and P^IV^ model compounds used above (due to already relatively short T_1_ values in **D**‐**G**), the 30–60 % gain in temporal resolution is still a welcome improvement, especially when considering that any free ligand present (either deliberately added in excess or liberated via exchange during catalytic turnover) would relax >90 % more quickly so that all ^31^P NMR spectra remain fully quantitative throughout the reaction.

Most importantly, no signs of spectral interference or cross‐reactivity in terms of ligand exchange or redox chemistry were observed for **D**‐**G** with **7**. Chemical shifts and peak linewidths were unaffected by the addition of [Cr(tmhd)_3_], and all NMR spectra remained unchanged with no observable precipitation or colour changes over at least 48 h at room temperature and 2 h at 50 °C (Tables S24–S26).

### Application of [Cr(tmhd)_3_] in catalytic reaction monitoring with multi‐nuclear FlowNMR spectroscopy

**Figure 10:**
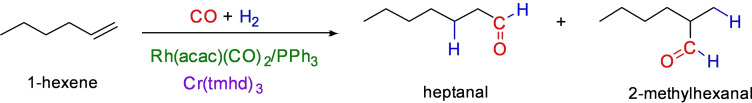
Hydroformylation of 1‐hexene [500 mM] catalysed by [Rh(acac)(CO)_2_]/PPh_3_ at 2.5 mM [Rh] in dry toluene in the presence of [Cr(tmhd)_3_] at 10 mM.

Hydroformylation reactions catalysed by phosphine‐modified Rh complexes represent an industrially important application of homogeneous catalysis that has been extensively studied by in‐situ IR[^66^, ^67^, ^68^] and more recently also by *operando* FlowNMR spectroscopy.[^19^, ^23^, ^69^] Due to the excess ligand used and multiple coordination equilibria present during turnover, causing a dynamic distribution of various Rh/PR_3_ intermediates of differing T_1_ values, the application of an inert PRA that shortens and equalises all spin‐lattice relaxation times would be particularly beneficial in such systems. The hydroformylation of 1‐hexene catalysed by [Rh(acac)(CO)_2_]/PPh_3_ at 50 °C under 10 bar of 1 : 1 CO/H_2_ using 1,3,5‐trimethoxybenzene (TMB) and TPOP as internal standards (section 1.2.1 Supporting Information) was thus investigated by ^1^H and ^31^P{^1^H} FlowNMR spectroscopy as described previously^[19]^ but here now in the presence of **7** (Figure 10).

In this example, ^1^H FlowNMR experiments provide information about the reaction progress (conversion, rate, chemo‐ and regioselectivity), and selective excitation ^1^H FlowNMR experiments serve to identify and quantify hydrido‐phosphine carbonyl complexes formed during the reaction (Figure S12).^[19]^ Interleaved ^31^P{^1^H} FlowNMR experiments allow insight into the speciation of phosphine‐modified Rh complexes, including those with acyl and hydride ligands. Despite sensitivity challenges due to limited ^31^P receptivity and significant *in‐flow* effects as a result of relatively long T_1_ values, the ^31^P distribution may be quantified against suitable internal ^31^P standards such as TPOP in this case (Figure S13).^[23]^

**Figure 11:**
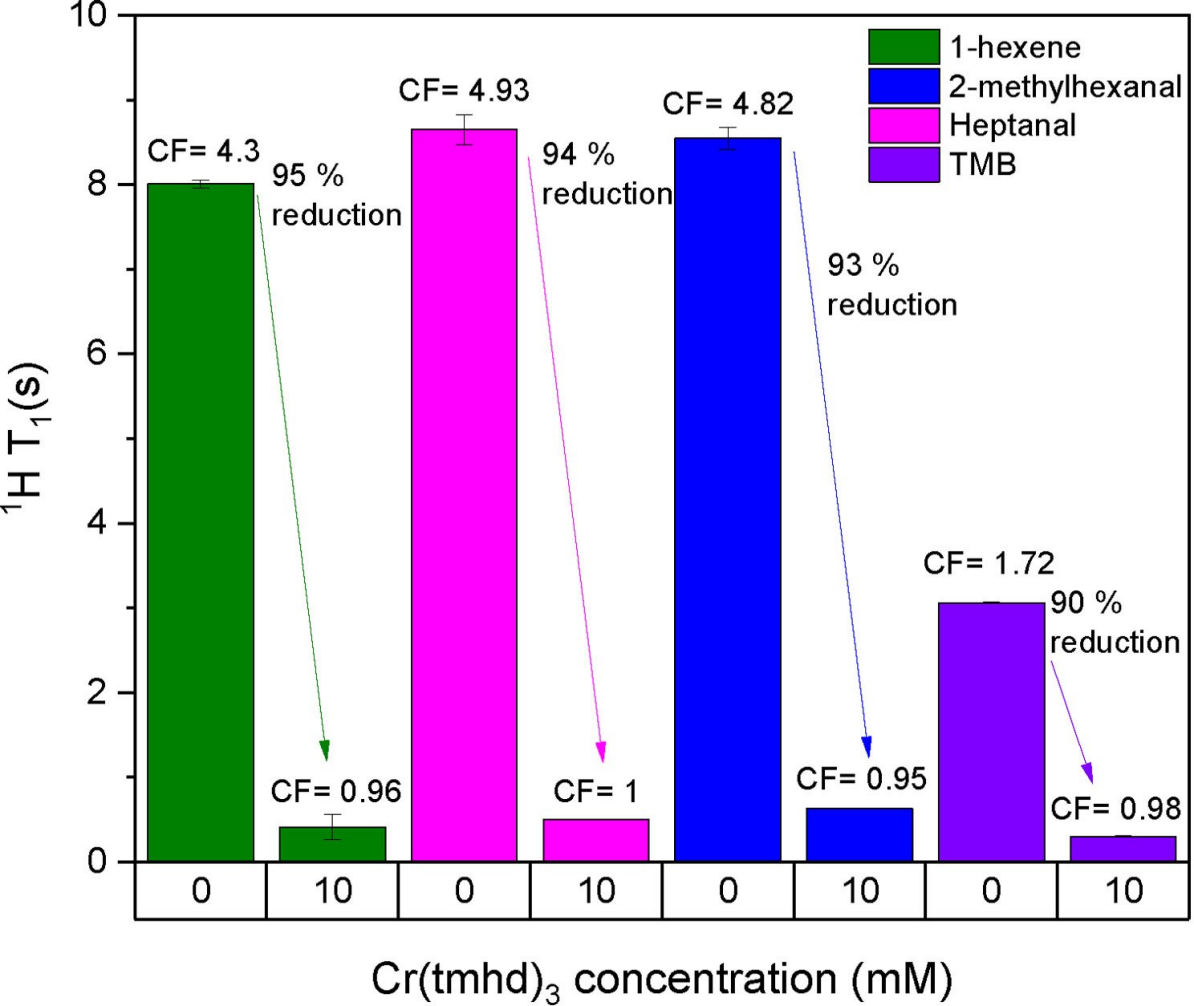
^1^H T_1_ values and flow correction factors (CF) of substrate, product and internal standard measured by inversion recovery experiments at 0 ° C under static conditions during the hydroformylation of 1‐hexene, with and without [Cr(tmhd)_3_] in dry toluene under 10 bar of syngas.

Without the use of **7**, the ^1^H spin‐lattice relaxation times of relevant substrate (terminal alkene) and product (aldehyde) peaks were in the order of 3–8 seconds under reaction conditions, resulting in integral correction factors (CF) of 1.2–4.9 at 4 mL/min (Tables S27 & S28). With the addition of 10 mM **7** the apparent T_1_ values decreased by >90 % to a range of 0.3–0.6 seconds to give almost negligible CFs of 0.95–1 (Tables S29 & S30), meaning that ^1^H FlowNMR spectra were essentially quantitative as acquired without the need of calibration (Figure 11). With S/N ratios in the order of 1000 sensitivity is generally not an issue in the ^1^H FlowNMR spectra on our equipment, so for flow experiments with **7** the number of scans was kept constant and the recycle time shortened to a minimum (section 2.2.2 of the Supporting Information) to reduce the ^1^H FlowNMR experiment time by 76 %. This not only makes reaction monitoring via ^1^H FlowNMR spectroscopy faster and easier to use, but also allows the quantification of transient intermediates for which static flow CFs cannot easily be obtained.

**Figure 12:**
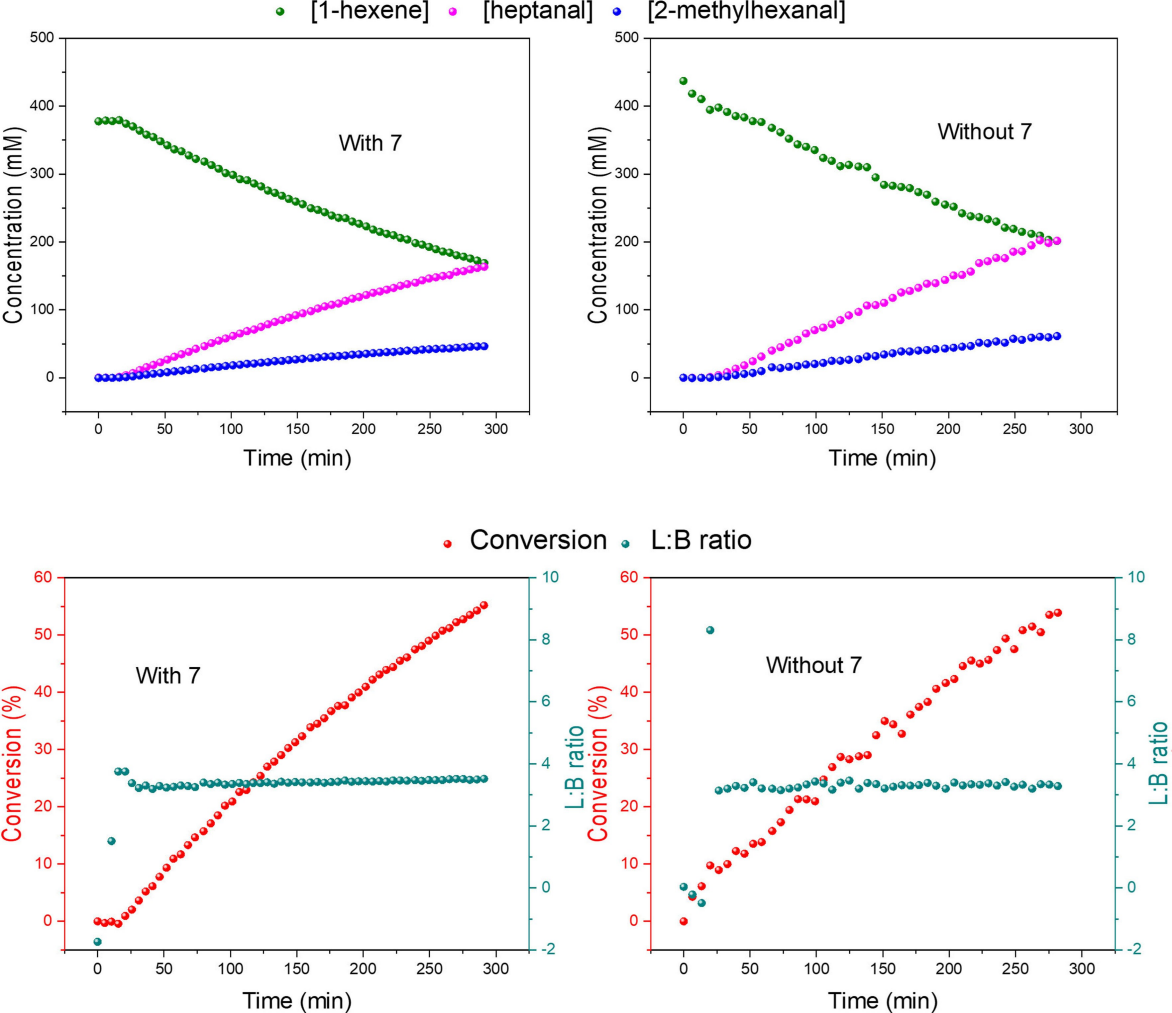
Consumption of 1‐hexene and formation of n‐heptanal and 2‐methylhexanal (upper) and conversion (lower) of the reaction as followed by ^1^H FlowNMR during the hydroformylation of 1‐hexene under 10 bar of CO/H_2_ (1 : 1) catalysed by [Rh(acac)(CO)_2_]=2.5 mM and PPh_3_=50 mM with 10 mM of [Cr(tmhd)_3_] in 22.4 mL of non‐deuterated toluene.

**Figure 13:**
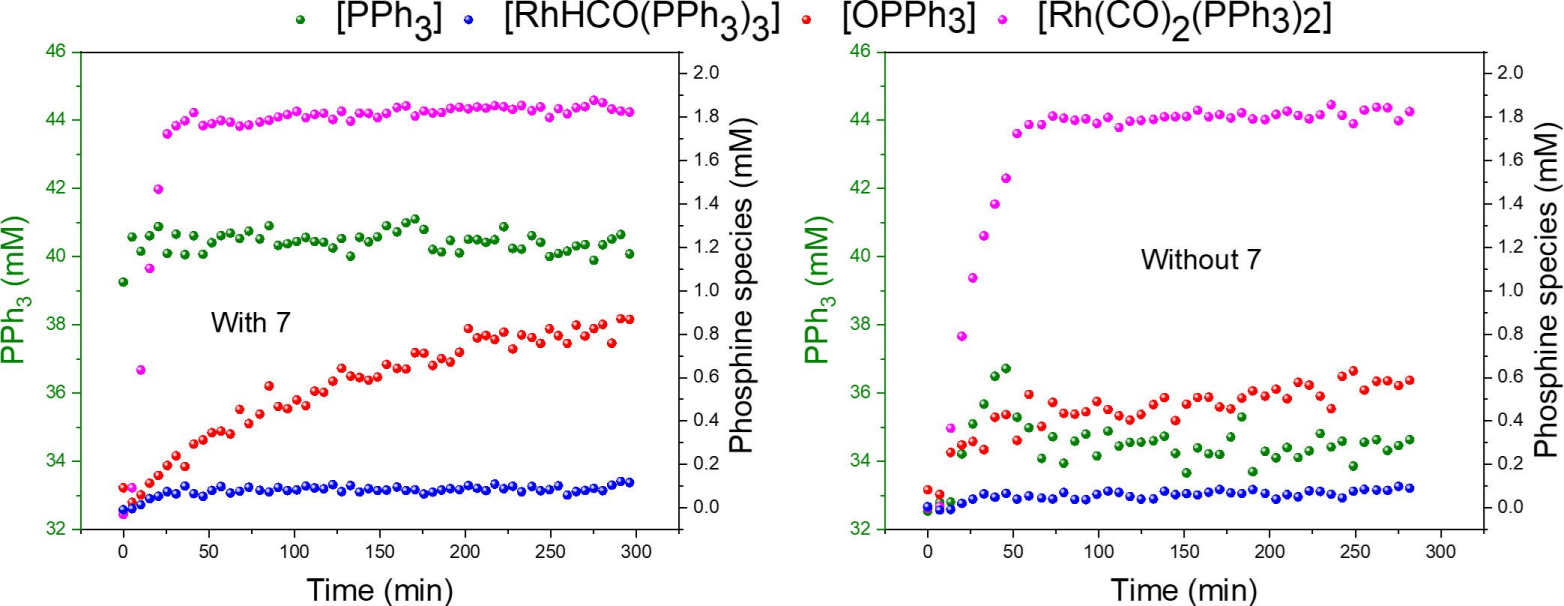
Concentration profile of phosphine species followed by ^31^P{^1^H} FlowNMR during the hydroformylation of 1‐hexene under 10 bar of CO/H_2_ (1 : 1) catalysed by [Rh(acac)(CO)_2_]=2.5 mM and PPh_3_=50 mM with 10 mM of [Cr(tmhd)_3_] in 22.4 mL of non‐deuterated toluene.

In the ^31^P{^1^H} FlowNMR spectra the S/N ratios for the various Rh/P intermediates, under our typical experimental conditions, were in the order of 10–100 in the absence of **7** due to their lower concentration and lower sensitivity than the substrates and products in the ^1^H domain. As expected from the results obtained with static mixtures of TPP, TPPO and TPOP (see above), the addition of **7** to the reaction reduced the ^31^P flow CFs from 3–11 s down to 1.7–2.7 s as a result of the 70–90 % increased spin‐lattice relaxation and magnetisation build‐up in flow (Tables S27–31). Due to the lower ^31^P signal intensities the effective shortening of the recycle time per scan afforded by **7** was used to accumulate more scans within the same experiment time to improve the average S/N by ∼30 %. Thus, while for ^31^P{^1^H} FlowNMR experiments integral CFs are still required for accurate quantification with an outer‐sphere PRA (at least under our conditions applied), the addition of 10 mM **7** significantly improved sensitivity of this useful NMR nucleus in flow (Tables S31–S35). Importantly, apart from slight differences in absolute concentrations due to experimental variation, comparison of the data from the same reaction with and without **7** showed identical rates, selectivities and speciation in the FlowNMR spectra acquired (Figures 12 & 13), demonstrating chemical compatibility with a transition‐metal catalysed reaction at elevated temperature and pressure with much improved data quality.

**Figure 14:**
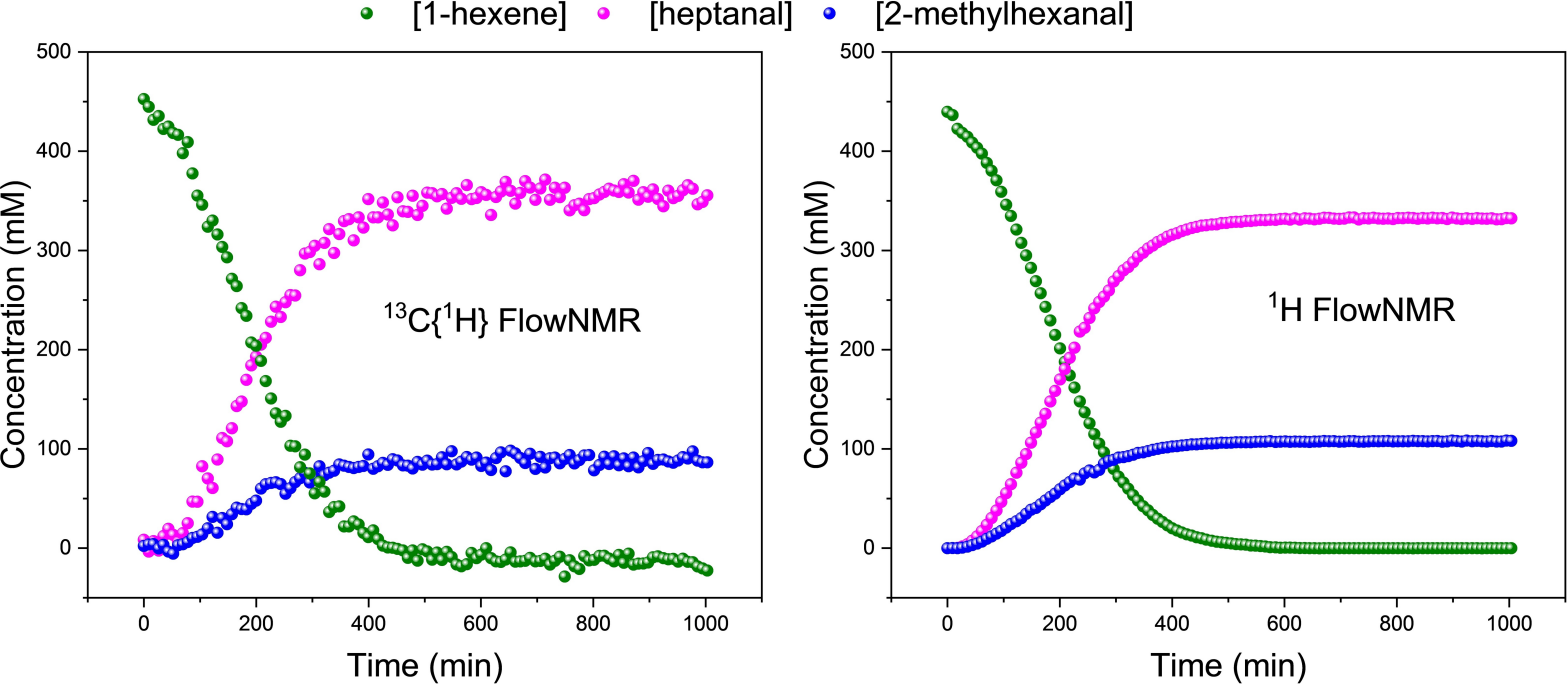
Consumption of 1‐hexene and formation of n‐heptanal and 2‐methylhexanal as followed by ^1^H (right) and ^13^C{^1^H} FlowNMR (left) during the hydroformylation of 1‐hexene under 10 bar of CO/H_2_ (1 : 1) catalysed by [Rh(acac)(CO)_2_]=2.5 mM and PPh_3_=7.5 mM with 10 mM of [Cr(tmhd)_3_] in 22.4 mL of non‐deuterated toluene.

Due to its low abundance, ^13^C{^1^H} FlowNMR has not been used to study hydroformylation reactions, although it is a potentially useful nucleus to allow the observation of Rh species without PR_3_ ligands and distinguish internal alkene double bond isomers formed during the reaction that may overlap in the ^1^H NMR spectra. Consistent with the effect of **7** observed on static ^13^C{^1^H} NMR data (see above), we saw a significant reduction in T_1_* values of 80 % for the ^13^C NMR signals of 1‐hexene, heptanal and 2‐methylhexanal that allowed reasonable ^13^C spectra with S/N values of 25–203 to be collected within 6 min at 4 mL/min under reaction conditions when just 10 mM **7** was added to the mixture. Although significant integral CFs of 9–14 were still required for accurate signal quantification (Table S36), the use of **7** allowed following the reaction progress and selectivity by ^13^C{^1^H} FlowNMR that matched the data from ^1^H FlowNMR very well (Figure 14).

## Conclusion

The range of spin‐lattice relaxation times across multiple NMR resonances in reactive mixtures has long bedevilled applied quantitative NMR spectroscopy. This issue is exacerbated in the context of kinetic measurements where fast acquisition is required, and further complicated by *in‐flow* effects in FlowNMR applications. Low receptivity heteronuclei with long T_1_ such as ^13^C and ^31^P particularly suffer from these effects, often to the point where sensitivity is so limited that they become practically useless. All of these issues can be addressed by the addition of a suitable paramagnetic relaxation agent that induces faster polarisation (reduction of limiting *in‐flow* effects) as well as faster relaxation (reduction of experiment time) to increase NMR sensitivity per unit time. This effect may be used to either shorten the experiment time to follow faster reactions (in case of abundant signal intensity) or accumulate more scans for increased sensitivity (in case of low signal intensity).

We have found octahedral Cr^III^ acetylacetonate complexes of *S*=3/2 to be effective for this purpose even though their paramagnetic moment is moderate by comparison with the lanthanide‐based contrast agents commonly used in MRI (Table 1). While with the simple acac ligand some ligand exchange reactions occurred in a mixture of triphenyl phosphine, phosphine oxide and phosphate at elevated temperature, the bulkier tmhd ligand proved equally effective but chemically inert up to 80 °C even with a variety of reactive transition metal complexes. [Cr(tmhd)_3_] is easily available (see 1.2.2 in the Supporting Information),[^63^, ^70^] bench‐stable, soluble in a range of organic solvents, and introduces minimal spectral interference due to its rigid structure and *D_3_* symmetry. At concentrations of only 10 mM, [Cr(tmhd)_3_] was found to reduce ^1^H and ^31^P spin‐lattice relaxation times by over 90 % and by 82 % for ^13^C without any adverse line broadening or negative impact on two‐dimensional correlation experiments. Data from variation of concentration and temperature support an outer‐sphere relaxation mechanism through non‐contact magnetic coupling between the three unpaired electrons of high‐spin Cr^III^ and the NMR‐active nuclei of surrounding analytes. The equalisation of T_1_ values induced by [Cr(tmhd)_3_] is particularly useful for making optimum use of spectrometer time by tight control of acquisition times.

**Table 1:** Key proprieties of the transition metal complexes analysed as candidates for PRA in heteronuclear FlowNMR spectroscopy.^[50]^

	[Co(acac)_3_] (**1**)	[Mn(acac)_3_] (**2**)	[Fe(acac)_3_] (**3**)	[Cr(acac)_3_] (**4**)	[Ni(acac)_2_]_3_ (**5**)	[Gd(tmhd)_3_] (**6**)
Stability	Air stable	Air stable	Air stable	Oxygen sensitive	Air stable	Hygroscopic
Solubility	Organic solvents	Organic solvents	Non‐polar organic solvents	Non‐polar organic solvents	Organic solvents & water	Organic solvents
Colour	Green	Black	Red	Deep maroon	Green	Colourless
Electron configuration	d^6^	d^4^	d^5^	d^3^	d^8^	f^7^
Unpaired electrons	4	4	5	3	2×3	7
Magnetic moment	4.13 μB	4.33 μB	5.23 μB	3.13 μB	6.21 μB	7.15 μB

The benefits of using a PRA such as [Cr(tmhd)_3_] for kinetic FlowNMR measurements has been demonstrated in a catalytic hydroformylation reaction where the PRA improved ^1^H and ^31^P NMR data quality (reduction of CFs and increase of S/N) and allowed for time‐resolved reaction monitoring by ^13^C{^1^H} FlowNMR at natural abundance. The relaxation afforded by [Cr(tmhd)_3_] produced fully quantitative ^1^H FlowNMR spectra which eliminates integral variations due to intentional or unintentional flow rate variations (as the sample will be always fully magnetised regardless of the exact residence time in the magnetic field), producing better quality of reaction profiles and allowing the quantification of fleeting intermediates for which flow correction factors may not be obtainable.

The utility of outer‐sphere PRAs such as [Cr(tmhd)_3_] in fast and quantitative (Flow)NMR applications likely extends to other *I*=1/2 nuclei such as ^29^Si, ^77^Se and ^15^N as well as slowly relaxing quadrupolar nuclei such as ^6^Li, and will be particularly useful in situations where low‐concentration species are targeted. Another advantage of chemically inert PRAs such as **7** is the elimination of T_1_ effects from diffusion analyses of chemical reactions by pulsed‐field gradient NMR methods which may influence observed diffusion coefficients of reacting species.[^71^, ^72^] As such, we believe the use of [Cr(tmhd)_3_] will benefit mechanistic studies of reactive systems in a wide range of areas, and also prove useful for low‐field NMR applications where sensitivity is more limited and flow effects are more pronounced than at high field.

## Experimental

### General

Toluene, tetrahydrofuran (THF) and acetone were purchased from Fisher Chemicals solvents in HPLC grade, with 99.9 % purity. All solvents were passed through 0.2 μm syringe filters (VWR 514‐0070) prior to flowing through the flow path. Dry toluene and dry THF were freshly distilled from sodium and potassium respectively under argon before every use. [Cr(acac)_3_], [Ni(acac)_2_]_3_, [Ga(tmhd)_3_], [Cr(tmhd)_3_], [Fe(acac)_3_], [Mn(acac)_3_], [Co(acac)_3_] were purchased from Sigma‐Aldrich and used without further purification. Triphenylphosphine (TPP), triphenylphosphate (TPOP), triphenylphosphine oxide (TPPO), Dicarbonyl(2,4‐pentanedionato)rhodium(I) [Rh(acac)(CO)_2_], 1,3,5‐trimethoxybenzene (TMB), Wilkinson's catalyst [HRh(CO)(PPh_3_)_3_], Crabtree's catalyst [(cod)Ir(PCy_3_)(py)]PF_6,_ BrettPhos Pd G_3_ catalyst, [Ru(dppe)(methallyl)_2_], phosphoric acid (H_3_PO_4_) solution (NMR reference standard 85 % in H_2_O), chromium(III) chloride hexahydrate (CrCl_3_ ⋅ 6H_2_O) and 2,2,6,6‐tetramethyl‐3,5‐heptanedione (Htmhd) were purchased in the highest purity available and used without further purification. 1‐hexene was purchased from Acros Organics, stirred over potassium overnight followed by a fractional distillation under argon over the same metal. Carbon monoxide (99.99 %) and hydrogen (99.95 %) gases were supplied by BOC.

Unless stated otherwise, all manipulations were carried out under an inert atmosphere of argon using standard Schlenk line and glove box techniques. All NMR samples were prepared by dilution in J. Young NMR tubes from concentrated stock solutions of TPP, TPOP, TPPO, metal complexes used as catalyst for homogeneous catalysed reactions and the metal complexes used as possible relaxing agents. The stock solutions were prepared in the glovebox and/or Schlenk line and stored in J. Young Schlenk flasks under argon atmosphere for all measurements and only exposed to air for experiments dedicated to observing its effect. For ^31^P{^1^H} NMR experiments, the samples contained 10 mM of the reference compound and 1–10 mM of the potential paramagnetic agent in 0.5 mL of non‐deuterated toluene if the reference compound was soluble or non‐deuterated THF if not. For ^13^C{^1^H} NMR experiments, the samples contained 150 mM of TPP, TPPO and TPOP, and 10–150 mM of [Cr(tmhd)_3_] in 0.5 mL of non‐deuterated toluene. Table 2 & 3 show the different samples prepared for ^31^P{^1^H} and ^13^C{^1^H} NMR experiments respectively.

**Table 2:** Overview of samples and concentrations used for ^31^P{^1^H} NMR experiments. *TPP, TPPO & TPOP samples were prepared in all the concentrations with all the PRAs shown in the table whereas [HRh(CO)(PPh_3_)_3_], [(cod)Ir(PCy_3_)(py)]PF_6,_ BrettPhos Pd G_3_ & [Ru(dppe)(methallyl)_2_] samples were prepared as 10 mM and 10 mM of [Cr(tmhd)_3_].

PRA	PRA concentration [mM]	Reference concentration [mM]
[Cr(acac)_3_]	1	10
	2	
	5	
	10	
	1	1
[Ni(acac)_2_]	10	10
[Gd(tmhd)_3_]	10	
[Cr(tmhd)_3_]	5	
	10*	
[Fe(acac)_3_]	10	
[Mn(acac)_3_]	10	
[Co(acac)_3_]	10	

**Table 3:** Overview of samples and concentrations used for ^13^C{^1^H} NMR experiments.

PRA	PRA concentration (mM)	TPP, TPPO & TPOP Concentration (mM)
[Cr(tmhd)_3_]	10	150
	150	

Hydroformylation of 1‐hexene was carried out in a 100 mL Büchi Miniclave pressure reactor made of glass and stainless‐steel lid connected to the flow NMR apparatus via 1/16” Swagelok connections. An annular micro‐annular gear pump (mzr‐6355 from HNP Mikrosysteme GmbH) or rotary tetra‐piston pump (Vici M6 HP) were used to circulate the reaction mixture through the 1/16” polyetheretherketone (PEEK, Upchurch Scientific) tubing with 0.76 mm i.d. connected to an InsightMR flow tube (Bruker) placed in the probe of the spectrometer.

UV‐vis data was recorded using a AvantesLight‐DH−S‐BAL light source with as AvaSpec‐2048 L photo‐spectrometer. NMR spectra were recorded on a Bruker 500 MHz Avance III HD equipped with a nitrogen‐cooled BBO Prodigy CryoProbe and a Bruker 500 MHz Avance III HD Ultrashield equipped with a room temperature BBO or BBFO probe. ^1^H NMR chemical shifts are referenced against TMS (99.5 % purity in CDCl_3_) and ^31^P NMR shifts are referenced to 85 % H_3_PO_4_ in H_2_O. The reaction monitoring software used was InsightMR, and data processing was performed with TopSpin 4.0.6 and DynamicCenter 2.5.6. Unless stated otherwise the NMR experiments were conducted at 298 K by default. The temperature on the NMR spectrometers was calibrated in 10 K steps from 278 K to 358 K (Table 4). For the temperature range between 278 K to 328 K a methanol‐d_4_ sample was used.^[73]^ The temperature range between 328 K to 358 K was calibrated with an ethylene glycol in DMSO‐d_6_ sample.^[74]^ To calculate the actual temperatures for all experiments the calibration data was fitted linearly using the Equation 2:

(2) $$T_{act}=0.9983\;x\;T_{set}+1.1683$$

**Table 4:** Temperature calibration for the temperature range between 273.2 K to 353.2 K using a methanol‐d_4_ and an ethylene glycol in DMSO‐d_6_ sample on the Bruker Avance II+ 500 MHz spectrometer.

$T_{set}$	273.2	278.2	283.2	288.2	293.2	298.2	303.2	308.2	313.2
$T_{act}$	273.90	278.90	283.89	288.88	293.87	298.86	303.85	308.84	313.84
$T_{set}$	318.2	323.2	328.2	333.2	338.2	343.2	348.2	353.2	
$T_{act}$	318.83	323.82	328.81	333.80	338.79	343.78	348.78	353.77	

### Methods

#### Hydroformylation reaction in flow

For hydroformylation reactions carried out in autoclave and monitored by FlowNMR spectroscopy see electronic Supporting Information and Ref. [19].

*Caution: carbon monoxide is a colourless, odourless and highly toxic gas – experiments should only be conducted in the presence of a calibrated CO sensor*.

The FlowNMR apparatus was flushed with laboratory grade toluene and then purged with argon for at least 10 min to remove traces of air and moisture. Triphenylphoshine (see Table 5)_,_ dicarbonyl(acetylacetonato)rhodium(I) (12.90 mg, 0.05 mmol), 1,3,5‐trimethoxybenzene (33.64 mg, 2 mmol), triphenylphosphate (73.09 mg, 0.22 mmol) and chromium(III) tris(2,2,6,6‐tetramethyl‐3,5‐heptanedionate) (120.36 mg, 0.2 mmol) were added to the pressure glass vessel together with a teflon‐coated stir bar followed by sealing of the autoclave with all tubing attached. The system was leak‐checked, vacuum‐argon cycled three times at room temperature and then kept under argon. The inlet of the flow tube was then moved into a separate Schlenk flask that contained dry toluene under argon, and the outlet to the waste bottle. Dry toluene was then pumped through the flow tube for 5 min to leave the transfer lines, pump and flow tube filled with dry solvent (6.4 mL). Thereafter, both flow tube ends were reconnected to the reactor which was topped up with dry toluene (15 mL) and 1‐hexene (84.16 mg, 1.25 mL, 10 mmol) against a flow of argon. The NMR tube and tip were then inserted into the spectrometer, stirring started and the reaction mixture was pumped through the system at 4 mL/min once all solids had fully dissolved. The reactor, heat exchanger and NMR probe were all heated or cooled down to the desired temperature; 50 °C, 10 °C and 0 °C respectively. Once the temperature had stabilised throughout the system the NMR spectrometer lock was turned off, shimmed on ^1^H peaks and tuned to proton and phosphorus. Spectra of the reagents were recorded both statically and at 4 mL/min. Acquisition parameters for interleaved ^1^H, selectively excited ^1^H and ^31^P{^1^H} NMR measurements were entered (details below) and the sequence commenced to start the FlowNMR reaction monitoring. After acquisition of at least one sequence of measurements the autoclave was firstly pressurised with 5 bar H_2_ followed by other 5 bar of CO to start the reaction. At the end of the reaction additional calibration spectra with and without flow were recorded before all heating was switched off, the flow stopped and the reactor carefully vented into the fumehood.

**Table 5:** Amounts of TPP charged within the autoclave reactor for each flow run reaction with different number of ligand equivalents.

	Ph_3_ loadings
[PPh_3_]/[Rh]=3	39.35 mg, 0.15 mmol
[PPh_3_]/[Rh]=20	262.30 mg, 1 mmol

#### Synthesis of [Cr(tmhd)_3_]

The synthesis was carried out as described in the procedure reported by Doyle et al.^[63]^

A solution of 3.0 g (0.011 mol) of chromium(III) chloride hexahydrate in a mixture of 25 mL of water and 65 mL of absolute ethanol was prepared in a 250 mL round‐bottomed flask. To this solution was added 20.0 g (0.33 mol) of urea, 5.0 g (0.027 mol) of 2,2,6,6‐tetramethyl‐3,5‐heptanedione (dipivaloylmethane), and a magnetic stirring bar. The flask was fitted with a reflux condenser, and the mixture heated to reflux at 85 °C with stirring for 24 h. During this time the solution changed from deep green to dark purple, and a dark‐coloured solid separated from the reaction mixture. The solution was cooled to room temperature and diluted with 100 mL of water. The crude solid product was collected by filtration and was washed with three 50 mL portions of water. After drying overnight in air at room temperature the solid was sublimed under vacuum (180 °C and 0.1 bar). Care should be exercised during the sublimation to prevent contamination of the dark purple product by a light green, slightly less volatile, contaminant. Since the crude is a light powder the sublimation procedure was improved by putting a pad of glass wool over the crude material below the cold section of the sublimation apparatus (4.5 g, 83 %). ^1^H NMR (500 MHz, Tol‐H_8_, 25 °C): δ 33 (s, broad, 3H, methine protons), 2.3 (s, broad, 54H, *tert*‐butyl protons). UV‐vis (Tol‐H_8_) λ_max_ (ϵ)=570 nm (56,760 M^−1^), 427 nm (80,540 M^−1^).

## Conflict of interest

The authors declare no conflict of interest.

1

## Supporting information

As a service to our authors and readers, this journal provides supporting information supplied by the authors. Such materials are peer reviewed and may be re‐organized for online delivery, but are not copy‐edited or typeset. Technical support issues arising from supporting information (other than missing files) should be addressed to the authors.

Supporting Information

## Data Availability

The data that support the findings of this study are available in the supplementary material of this article.
