# Supplementary material for: Paramagnetic Relaxation Agents for Enhancing Temporal Resolution and Sensitivity in Multinuclear FlowNMR Spectroscopy
Source: Chemistry. 2023 May 17;29(38):e202300215. doi: 10.1002/chem.202300215 (PMC10962566; doi:10.1002/chem.202300215)
Supplement: Supplementary file 1 — Supporting Information [file CHEM-29-0-s001.pdf]

Chemistry–A European Journal

Supporting Information

# Paramagnetic Relaxation Agents for Enhancing Temporal Resolution and Sensitivity in Multinuclear FlowNMR Spectroscopy

Alejandro Bara-Estaún, Marie C. Harder, Catherine L. Lyall, John P. Lowe, Elizaveta Suturina,* and Ulrich Hintermair*

**Contents**

1. Analysis ........ 2
1.1 NMR acquisition parameters for static tube samples ........ 2
2.1.1 $T_1$ inversion recovery experiments ........ 2
2.1.2 One-dimensional experiments ........ 3
2.1.3 Two-dimensional experiments ........ 4
2.2 FlowNMR acquisition parameters ........ 5
2.2.1 Without PRA ........ 5
1.2.2. With PRA ........ 5
2. Quantification of FlowNMR data ........ 7
2.1 Correction factors ........ 7
3. Catalysis metrics ........ 8
4. Build-up magnetisation ........ 8
5. Additional data ........ 9
6. References ........ 28

# 1. Analysis

## 1.1 NMR acquisition parameters for static tube samples

### 2.1.1 $T_1$ inversion recovery experiments

- $^{13}C$ $T_1$ experiments with $^1H$ decoupling. Longer delay times were used for samples in the absence of a PRA, and shorter delays with the PRA added.

| | $^{13}C\{^1H\}$ $T_1$ NMR ($t_1$irig) without PRA | $^{13}C\{^1H\}$ $T_1$ NMR ($t_1$irig) with PRA |
|---|---|---|
| Number of scans (NS) | 8 | 8 |
| Delay time ($D_1$) | 125 s | 20 s |
| Receiver gain (RG) | 203 | 203 |
| Centre of spectrum ($O_1P$) | 100 ppm | 100 ppm |
| Spectral width (SW) | 250 ppm | 250 ppm |
| Experimental time (Expt) | 3 h 27 min 30 s | 1h 32 min 50 s |
| Acquisition time (AQ) | 0.75 s | 0.75 s |
| Loop counter ($L_4$) | 8 | 8 |
| Size of FID (TD) | 16384, 8 | 16384, 8 |
| Variable delay list (Vdlist) | 0.01, 0.5, 2.0, 10.0, 15.00, 30.0, 60.0, 125.0 | 0.01, 0.25, 0.5, 1.0, 5.0, 10.0, 15.0, 20.0 |

- $^{31}P$ $T_1$ experiments carried out with and without proton decoupling. Longer delay times were used for samples in the absence of a PRA, and shorter delays with the PRA added.

| | $^{31}P\{^1H\}$ $T_1$ NMR ($t_1$irig) | $^{31}P$ $\{^1H\}$ $T_1$ NMR ($t_1$ir) | $^{31}P$ $T_1$ NMR ($t_1$ir) |
|---|---|---|---|
| Number of scans (NS) | 8 | 8 | 8 |
| Delay time ($D_1$) | 15 s | 15 s | 100 s |
| Receiver gain (RG) | 203 | 203 | 203 |
| Centre of spectrum ($O_1P$) | 0 ppm | 0 ppm | 0 ppm |
| Spectral width (SW) | 100 ppm | 100 ppm | 100 ppm |
| Experimental time (Expt) | 32 min 0 s | 32 min 0 s | 1 h 21 min 6 s |
| Acquisition time (AQ) | 0.70 s | 0.70 s | 0.70 s |
| Loop counter ($L_4$) | 8 | 8 | 8 |
| Size of FID (TD) | 16384, 8 | 16384, 8 | 16384, 8 |
| Variable delay list (Vdlist) | 0.01, 0.1, 0.5, 1.0, 3.0, 5.0, 10.0, 15.0 | 0.01, 0.1, 0.5, 1.0, 3.0, 5.0, 10.0, 15.0 | 0.01, 0.5, 2.0, 6.0, 10.0, 15.0, 20.0, 35.0 |

- $^1H$ $T_1$ experiments.

| | $^1H$ $T_1$ NMR ($t_1$irig) |
|---|---|
| Number of scans (NS) | 8 |
| Delay time ($D_1$) | 45 s |
| Receiver gain (RG) | 203 |
| Centre of spectrum ($O_1P$) | 6.175 ppm |
| Spectral width (SW) | 20 ppm |
| Experimental time (Expt) | 1h 35 min 25 s |
| Acquisition time (AQ) | 0.82 s |
| Loop counter ($L_4$) | 8 |
| Size of FID (TD) | 16384, 8 |
| Variable delay list (Vdlist) | 0.01, 0.5, 3.0, 7.0, 10.0, 15.0, 30.0, 45.0 |

### 2.1.2 One-dimensional experiments

| | $^1H$ NMR (zg30) | $^{13}C\{^1H\}$ NMR (zgpg30) |
|---|---|---|
| Number of scans (NS) | 16 | 128 |
| Dummy scans (DS) | - | 4 |
| Delay time ($D_1$) | 60 s | 4 s |
| Receiver gain (RG) | 4.5 | 203 |
| Centre of spectrum ($O_1P$) | 6.175 | 100 ppm |
| Spectral width (SW) | 40 ppm | 250 ppm |
| Experimental time (Expt) | 16 min 26 s | 11 min 21 s |
| Acquisition time (AQ) | 1.64 s | 1.10 s |
| Constant used in pulsed programs (cnst21) | - | - |
| Constant used in pulsed programs (cnst55) | - | - |

| | $^{31}P\{^{1}H\}$ NMR (zgig60) | $^{31}P\{^{1}H\}$ NMR (zgpg60) |
|---|---|---|
| Number of scans (NS) | 40 | 40 |
| Dummy scans (DS) | 4 | 4 |
| Delay time ($D_1$) | 35 s | 35 s |
| Receiver gain (RG) | 203 | 203 |
| Centre of spectrum ($O_1P$) | 50 ppm | 50 ppm |
| spectral width (SW) | 400 ppm | 400 ppm |
| Experimental time (Expt) | 23 min 47 s | 23 min 38 s |
| Acquisition time (AQ) | 0.40 s | 0.40 s |

### 2.1.3 Two-dimensional experiments

| | $^{1}H$-$^{13}C$ HMBC NMR (hmbcetgpl3nd) | $^{1}H$-$^{31}P$ HMBC NMR (hmbcgpndqf) |
|---|---|---|
| Number of scans (NS) | 16 | 8 |
| Dummy scans (DS) | 16 | 16 |
| Delay time ($D_1$) | 1.33 s | 1.5 s |
| Receiver gain (RG) | 203 | 203 |
| Centre of spectrum ($O_1P$) | 5.50, 110 ppm | 6.125, 25 ppm |
| Spectral width (SW) | 20, 400 ppm | 20, 300 ppm |
| Experimental time (Expt) | 55 min 38s | 28 min 14 s |
| Acquisition time (AQ) | 0.40 s | 0.40 s |

## 2.2 FlowNMR acquisition parameters

### 2.2.1 Without PRA

$^{1}H$, selectively excited $^{1}H$, and $^{31}P\{^{1}H\}$ NMR experiments were interleaved in each cycle and continuously executed every 5 minutes 22 seconds until the end of the reaction.

| | $^{1}H$ NMR (zg30) | Selective excitation $^{1}H$ NMR (seldpfgse_calc.ptg) | $^{31}P\{^{1}H\}$ NMR (zgpg60) |
|---|---|---|---|
| Number of scans (NS) | 16 | 32 | 320 |
| Delay time ($D_1$) | 1 s | 1 s | 0.1 s |
| Receiver gain (RG) | 9 | 203 | 203 |
| Centre of spectrum ($O_1P$) | 4.7 | -5 ppm | 50 ppm |
| Spectral width (SW) | 40 ppm | 20 ppm | 400 ppm |
| Experimental time (Expt) | 42 s | 1 min 37 s | 3 min 03 s |
| Acquisition time (AQ) | 1.64 s | 2 s | 0.40 s |
| Constant used in pulsed programs (cnst21) | - | -8 | - |
| Constant used in pulsed programs (cnst55) | - | 5 | - |

Static calibration spectra were recorded with the same acquisition parameters but with increased delay times as follows:

- $^{1}H$ NMR spectra ➔ $D_1$ = 60 s
- Selective excitation $^{1}H$ NMR spectra ➔ $D_1$ = 15 s
- $^{31}P\{^{1}H\}$ NMR (zgig60) spectra ➔ $D_1$ = 90 s

### 1.2.2. With PRA

The acquisition parameters were optimised when using the PRA due to quicker relaxation of the nuclei. For nuclei where signal-to-noise (S/N) is sufficient such us $^{1}H$ the NS was kept constant and the experimental time shorted by reducing $D_1$ and AQ. For measurements where more sensitivity was needed ($^{31}P$ &$^{1}H$ SelEx) the NS was increased while shortening $D_1$ and AQ but maintaining the same experimental time. $D_1$ was shortened to 0.1s as the minimum

possible value while AQ was modified observing the FID of the experiments with the old parameters. Different values were tried for each experiment until incipient truncation of the FID causing spectral distortions to indicate minimum AQ.

$^{1}H$, selectively excited $^{1}H$, and $^{31}P\{^{1}H\}$ NMR experiments were interleaved in each cycle and continuously executed every 4 minutes 50 seconds until the end of the reaction.

| | $^{1}H$ NMR (zg30) | Selective excitation $^{1}H$ NMR (seldpfgse_calc.ptg) | $^{31}P\{^{1}H\}$ NMR (zgpg60) |
|---|---|---|---|
| Number of scans (NS) | 16 | 152 | 436 |
| Delay time ($D_1$) | 0.1 s | 0.1 s | 0.1 s |
| Receiver gain (RG) | 1.6 | 203 | 203 |
| Centre of spectrum ($O_1P$) | 4.7 | -5 ppm | 50 ppm |
| Spectral width (SW) | 40 ppm | 20 ppm | 400 ppm |
| Experimental time (Expt) | 10 s | 1 min 37 s | 3 min 03 s |
| Acquisition time (AQ) | 0.5 s | 0.5 s | 0.25 s |
| Constant used in pulsed programs (cnst21) | - | -8 | - |
| Constant used in pulsed programs (cnst55) | - | 5 | - |

| | $^{13}C\{^{1}H\}$ NMR (zgpg30) |
|---|---|
| Number of scans (NS) | 400 |
| Delay time ($D_1$) | 0.1 s |
| Receiver gain (RG) | 203 |
| Centre of spectrum ($O_1P$) | 100 ppm |
| Spectral width (SW) | 40 ppm |
| Experimental time (Expt) | 5 min 45 s |
| Acquisition time (AQ) | 0.7 s |
| Constant used in pulsed programs (cnst21) | - |
| Constant used in pulsed programs (cnst55) | - |

# 2. Quantification of FlowNMR data

## 2.1 Correction factors

In-flow effects resulting from different degrees of pre-magnetisation may be corrected for by comparing integral values from flow spectra with static reference measurements. A correction factor is then calculated for each peak comparing the integral at static and flow conditions and used for quantifying the data (**Figure S1 & Table S5**).

$$\mathrm{CF} = \frac{I_{\mathrm{static}}}{I_{\mathrm{flow}}}$$

$$I_{\mathrm{corrected}} = \mathrm{CF} \text{ x } I$$

$$C_{\mathrm{compound}} = \frac{I_{\mathrm{compound\ corrected}}}{I_{\mathrm{internal\ standard\ corrected}}} \text{ x } \frac{\mathrm{N}_{\mathrm{internal\ standard}}}{\mathrm{N}_{\mathrm{compound}}} \text{ x } \mathrm{C}_{\mathrm{internal\ standard}}$$

***Equation S1**. Formulas used to calculate the concentrations of each compound during the reaction. I=peak integral, CF= correction factor, C= concentration and N=number of nuclei contributing to the peak.*

Selectively excited $^{1}$H spectra were recorded at a much higher RG settings. Thus, to allow these experiments to be quantitative relative to other signals detected without selective excitation, a relative integral value (RIV) was calculated to account for the difference in receiver gains (**Figure S2**).[1]

$$RIV = \frac{I_{\mathrm{hydride}}\ x\ RG_{normal}\ x\ RGCF}{RG\ hydride}$$

$$[Hydride] = RIV x\ \frac{C_{internal\ \mathrm{standard}}}{\frac{I_{internal\ standard}}{N_{internal\ standard}}}$$

***Equation S2.** Formulas used to calculate the concentrations of hydride species. I=peak integral, CF= correction factor, C= concentration and N=number of nuclei causing the peak.*

## 3. Catalysis metrics

Conversion was calculated as:

*% = 100 – ([1-hexene]/[1-hexene]$_0$ * 100)*

Chemoselectivity towards hydroformylation was calculated as:

*% = ([n-heptanal] +[2-methylhexanal])/ ([h-heptanal] + [2-methylhexanal] + [2- and 3-hexene]) * 100*

Linear-to-branched product ratio was calculated as:

*L:B = ([n-heptanal] / [2-methylhexanal])*

## 4. Build-up magnetisation

Assuming a monoexponential build-up of magnetisation (**Equation S1**).[2]

***Equation S3.*** *The build-up of longitudinal spin magnetization, after the magnetic field is turned on.*

$$\frac{M(t)}{M_{eq}} = \left(1 - \exp\left[-\frac{t - t_0}{T_1}\right]\right) * 100\%$$

## 5. Additional data

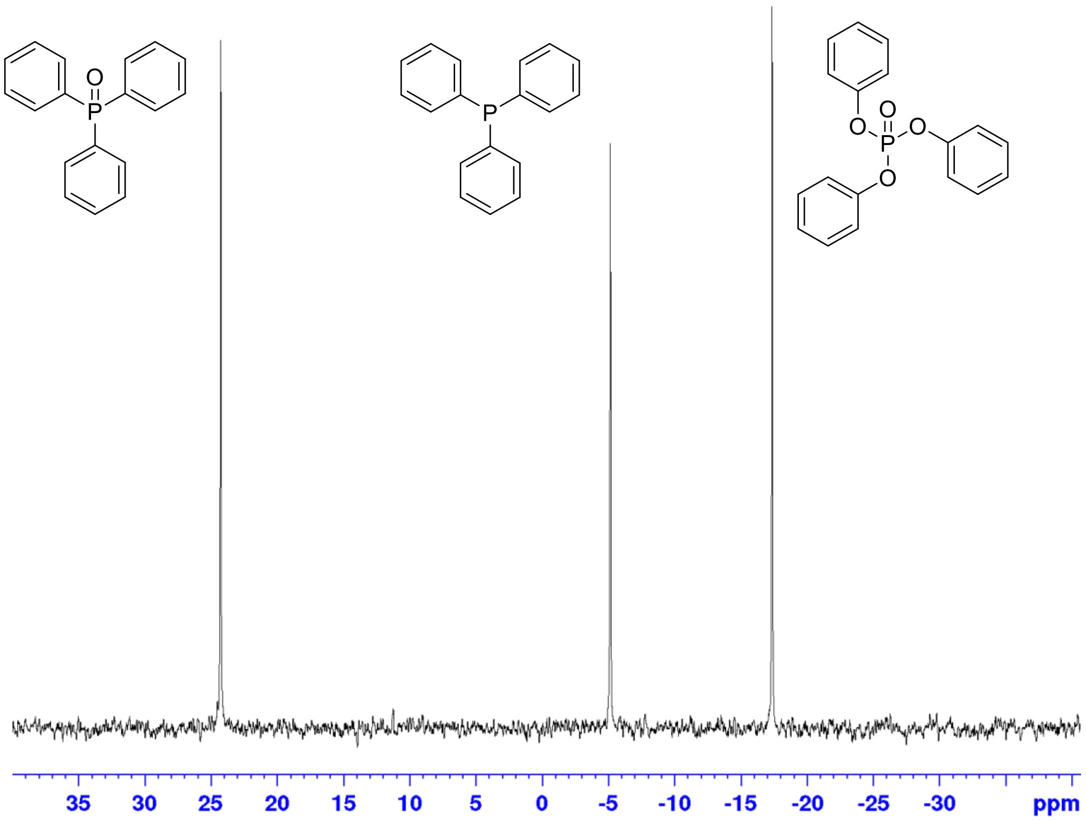


***Figure S1.*** *Structures of the three phosphorous model compounds and $^{31}P\{^{1}H\}$ spectrum of an equimolar mixture at 10 mM each in 0.5 mL of non-deuterated, dry toluene recorded at 298 K under Argon. Number of scans (NS) = 16, Flip angle (FA) = 30°, Delay time ($D_1$) = 2 sec, Acquisition time (AQ) = 0.81 sec, Centre of spectrum ($O_1P$) = 0 ppm, Spectral width (SW) = 200 ppm.*

***Table S5****. Comparison of chemical shift, peak linewidth and integral of TPP, TPPO and TPOP as recorded by $^{31}P\{^{1}H\}$ spectroscopy at 298 K under Ar before and after heating up the sample to 353 K for one hour.*

| **Heating** | **Species** | **$\delta$ (ppm)** | **$\Delta\delta$ (ppm)** | **FWHM (Hz)** | **Absolute integral (a.u.)** |
|---|---|---|---|---|---|
| **Before** | TPPO | 24.30 | - | 1.04 | 39.33 |
| **After** | TPPO | 24.35 | 0.054 | 4.02 | 91.42 |
| **Before** | TPP | -5.13 | - | 1.20 | 30.43 |
| **After** | TPP | -5.11 | 0.022 | 1.69 | 67.77 |
| **Before** | TPOP | -17.38 | - | 0.50 | 45.86 |
| **After** | TPOP | -17.35 | 0.028 | 1.35 | 109.98 |

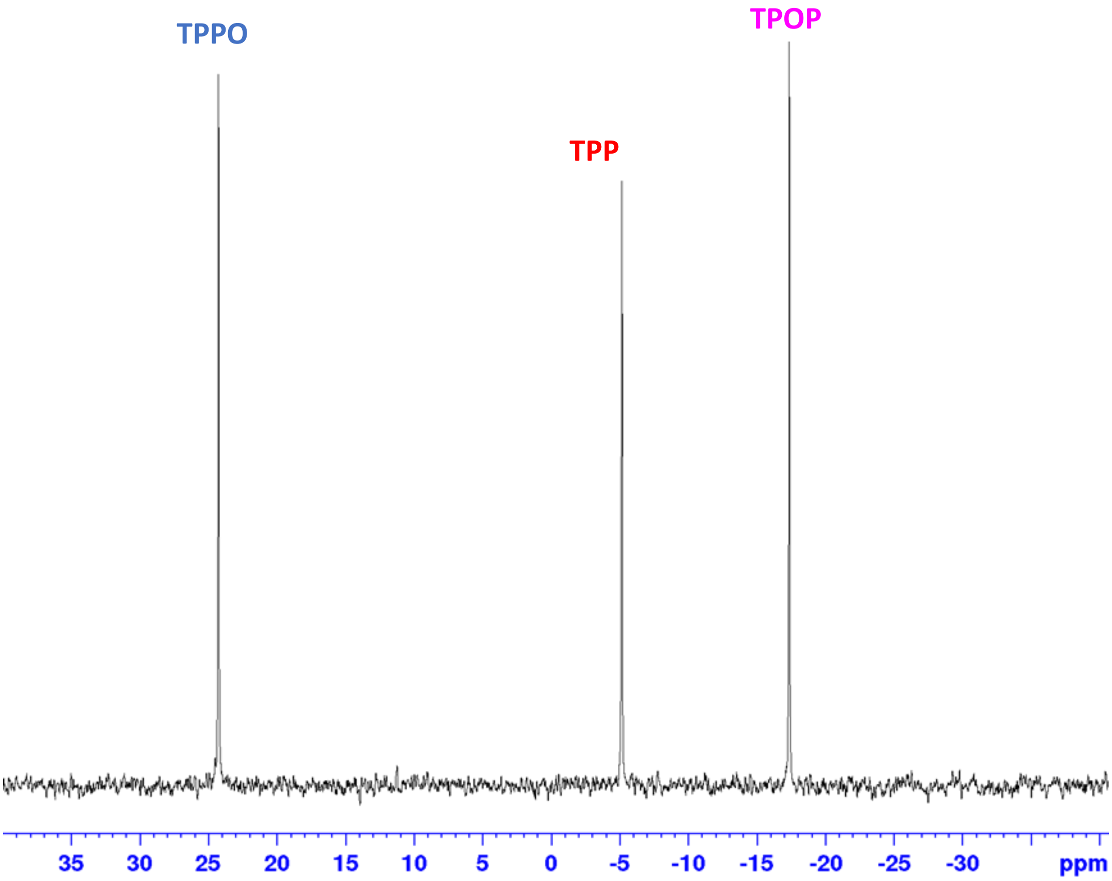


***Figure S2.*** *$^{31}P\{^{1}H\}$ spectrum of a sample containing 10 mM of TPP, TPPO and TPOP in 0.5 mL of non-deuterated dry toluene recorded at 298 K under Ar after having heated the sample for 1 h to 353 K.*

***Table S6.*** *$^{31}P$ $T_1$ values of TPP, TPPO and TPOP as measured by inversion recovery experiments at 298 K under Ar or air in a sample containing 10 mM of TPP, TPPO and TPOP in 0.5 mL of dry non-deuterated toluene.*

| Sample at 298 K | $T_1$ TPPO (s) | $T_1$ TPP (s) | $T_1$ TPOP (s) |
|---|---|---|---|
| **In Argon** | 5.00 ± 0.13 | 18.80 ± 0.27 | 4.31 ± 0.04 |
| **In Air** | 4.84 ± 0.12 | 17.50 ± 0.58 | 4.21 ± 0.12 |

***Table S7.*** *$^{31}P$ $T_1$ values of TPP, TPPO and TPOP at 10mM as measured by inversion recovery experiments at 298 K under Ar after heating to 353 K for one hour.*

| Sample at 298 K | | $T_1$(TPPO) (s) | Δ | $T_1$(TPP) (s) | Δ | $T_1$(TPOP) (s) | Δ |
|---|---|---|---|---|---|---|---|
| **1** | **Before Heating** | 5.00 ± 0.13 | - | 18.80 ± 0.27 | - | 4.31 ± 0.04 | - |
| | **After Heating** | 4.71 ± 0.27 | -5.8% | 17.80 ± 1.16 | -5.3% | 4.25 ± 0.12 | -1.4% |

***Table S8.*** *PRAs tested with chemical shift, linewidth, line broadening, peak intensity, and integral areas for the phosphorus species TPPO, TPP and TPOP respectively.*

| Complex | Species | Frequency (Hz) | Width (Hz) | Line broadening | Integral area (a.u) |
|---|---|---|---|---|---|
| none | **TPPO** | 24.28 | 0.67 | 1.00 | 102.17 |
| | **TPP** | -5.15 | 1.32 | 1.00 | 94.51 |
| | **TPOP** | -17.37 | 0.65 | 1.00 | 108.18 |
| [Cr(acac)$_3$] (4) | **TPPO** | 24.86 | 2.14 | 3.17 | 127.48 |
| | **TPP** | -5.085 | 2.16 | 1.63 | 98.76 |
| | **TPOP** | -17.33 | 1.49 | 2.31 | 116.55 |
| [Ni(acac)$_2$]$_3$ (5) | **TPPO** | - | - | - | - |
| | **TPP** | - | - | - | - |
| | **TPOP** | -17.28 | 11.40 | 17.67 | 754.16 |
| [Gd(tmhd)$_3$] (6) | **TPPO** | - | - | - | - |
| | **TPP** | -4.00 | 15.50 | 11.72 | 762.09 |
| | **TPOP** | -16.35 | 9.30 | 14.42 | 216.73 |
| [Fe(acac)$_3$] (3) | **TPPO** | 24.97 | 15.50 | 22.99 | 81.48 |
| | **TPP** | -4.47 | 3.46 | 2.62 | 117.13 |
| | **TPOP** | -16.79 | 1.70 | 2.64 | 127.98 |
| [Mn(acac)$_3$] (2) | **TPPO** | - | - | - | - |
| | **TPP** | -4.73 | 6.68 | 5.05 | 422.54 |
| | **TPOP** | -16.97 | 6.13 | 9.50 | 451.73 |
| [Co(acac)$_3$] (1) | **TPPO** | 24.34 | 8.45 | 12.53 | 148.78 |
| | **TPP** | -5.13 | 9.57 | 7.23 | 133.80 |
| | **TPOP** | -17.38 | 1.56 | 2.43 | 116.96 |

***Table S9***. *$^{31}$P $T_1$ values of TPP, TPPO and TPOP as measured by inversion recovery experiments at 298 K under Ar in samples containing 10 mM of TPP, TPPO and TPOP and 0, 1, 2, 5 and 10 mM of [Cr(acac)$_3$] in 0.5 mL of dry non-deuterated toluene.*

| **[Cr(acac)$_3$] (mM)** | **$T_1$ TPPO (s)** | **$T_1$ reduction (%)** | **$T_1$ TPP (s)** | **$T_1$ reduction (%)** | **$T_1$ TPOP (s)** | **$T_1$ reduction (%)** |
|---|---|---|---|---|---|---|
| 0 | 5.00 ± 0.13 | - | 18.80 ± 0.27 | - | 4.31 ± 0.04 | - |
| 1 | 3.32 ± 0.23 | 33.60 | 9.55 ± 0.57 | 49.20 | 3.27 ± 0.04 | 24.13 |
| 2 | 2.51 ± 0.06 | 49.80 | 6.20 ± 0.09 | 67.02 | 2.78 ± 0.02 | 35.50 |
| 5 | 1.27 ± 0.06 | 74.60 | 2.76 ± 0.15 | 85.32 | 1.70 ± 0.06 | 60.56 |
| 10 | 0.68 ± 0.01 | 86.40 | 1.48 ± 0.07 | 92.13 | 1.06 ± 0.02 | 75.41 |

***Table S10***. *$^{31}$P $T_1$ values of TPP, TPPO and TPOP as measured by inversion recovery experiments at 298 K under air in samples containing 10 mM of TPP, TPPO and TPOP and 0, 1, 2, 5 and 10 mM of [Cr(acac)$_3$] in 0.5 mL of wet non-deuterated toluene.*

| **[Cr(acac)$_3$] (mM)** | **$T_1$ TPPO (s)** | **$T_1$ reduction (%)** | **$T_1$ TPP (s)** | **$T_1$ reduction (%)** | **$T_1$ TPOP (s)** | **$T_1$ reduction (%)** |
|---|---|---|---|---|---|---|
| 0 | 4.84 ± 0.12 | - | 17.5 ± 0.58 | - | 4.21 ± 0.12 | - |
| 1 | 3.06 ± 0.23 | 36.78 | 8.75 ± 0.15 | 50.00 | 3.26 ± 0.16 | 22.57 |
| 2 | 4.46 ± 0.99 | 7.85 | 5.38 ± 0.65 | 69.26 | 2.74 ± 0.09 | 34.92 |
| 5 | 1.21 ± 0.02 | 75.00 | 2.86 ± 0.09 | 83.66 | 1.70 ± 0.04 | 59.62 |
| 10 | 0.83 ± 0.04 | 82.85 | 1.44 ± 0.03 | 91.77 | 1.06 ± 0.01 | 74.82 |

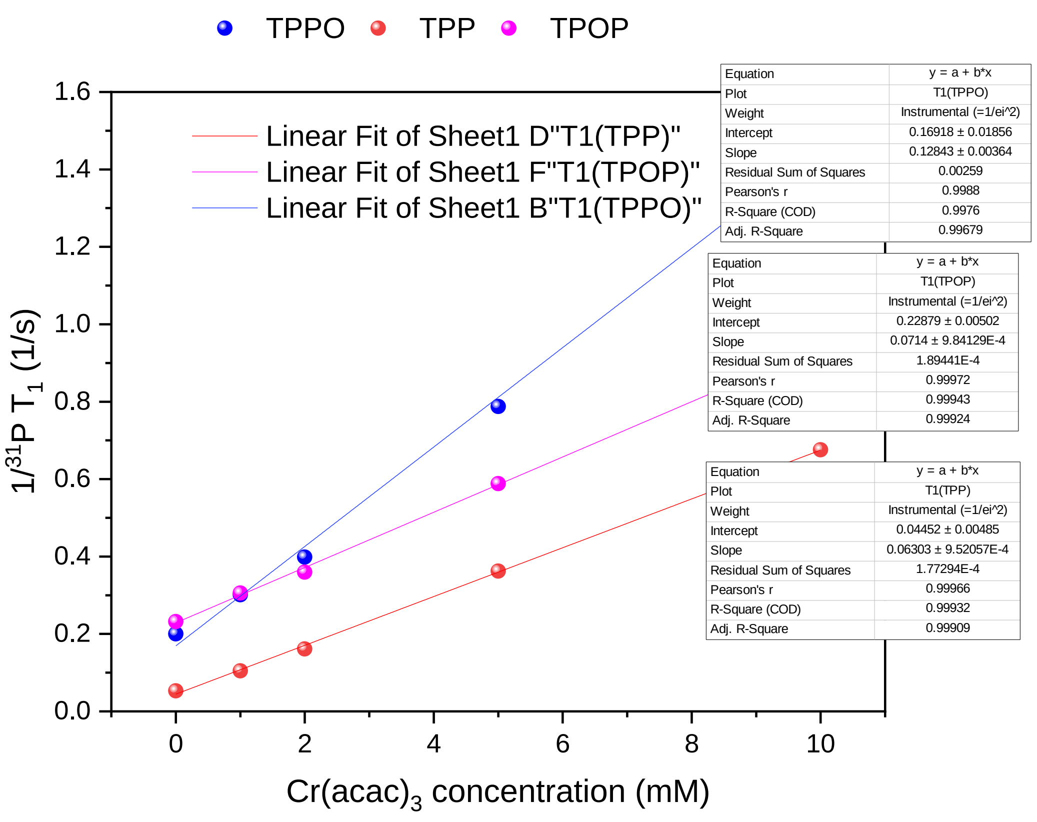


***Figure S3***. *Linear fits and equations for the 1/T1 of the phosphorus containing species with 0, 1, 2, 5 and 10 mM of [Cr(acac)$_3$] in dry toluene under Argon.*

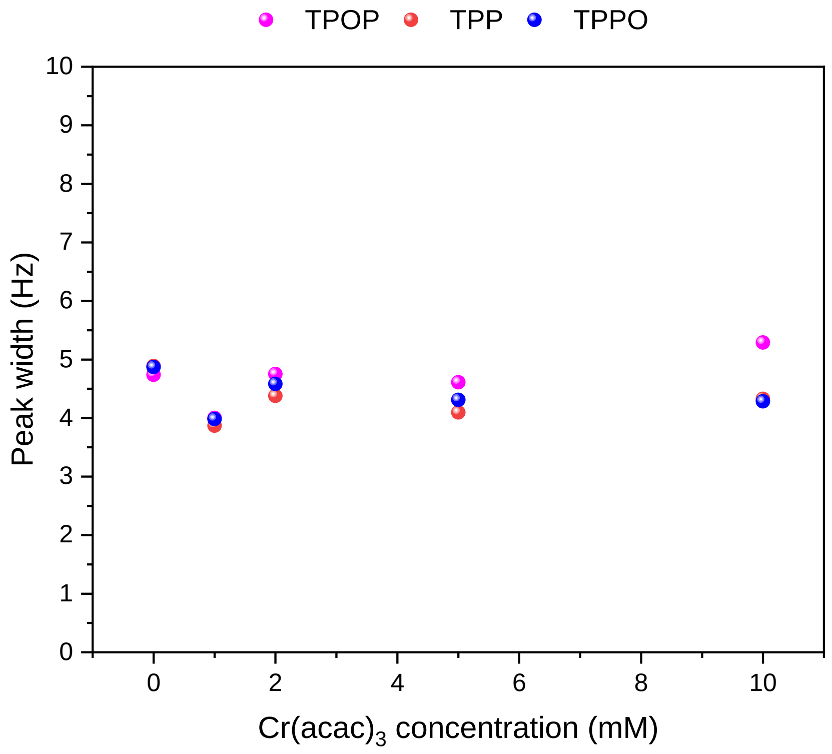


***Figure S4***. *Peak width values of TPP, TPPO and TPOP as measured by $^{31}P\{^1H\}$ NMR spectroscopy at 298 K in samples containing 10 mM of each analyte with 0, 1, 2, 5 and 10 mM of [Cr(acac)$_3$] in dry toluene under Argon.*

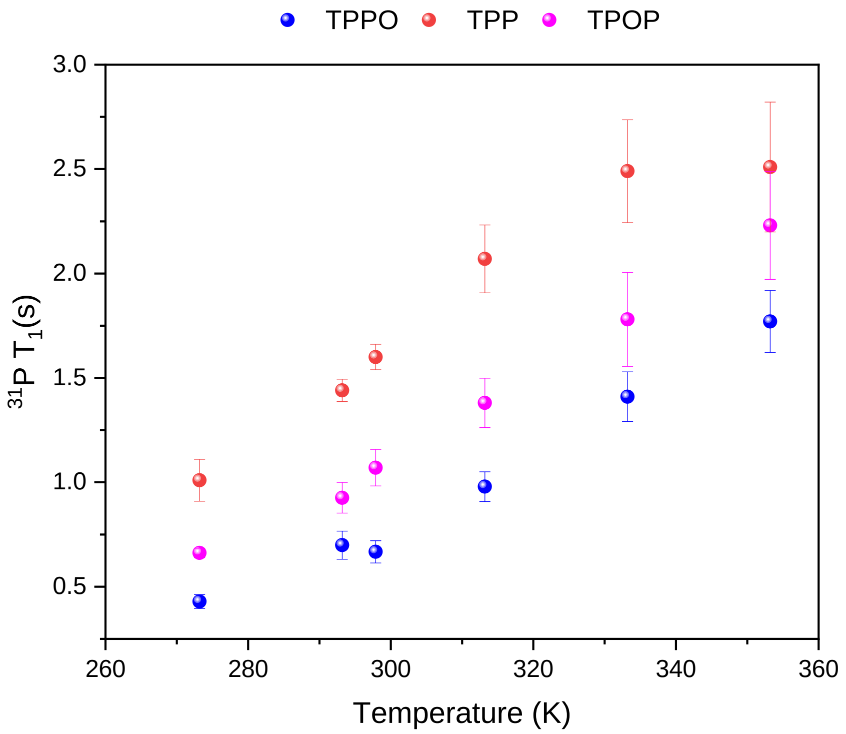


***Figure S5***. *[31]P $T_1$ of TPPO (blue), TPP (red) and TPOP (magenta) as a function of temperature at which $T_1$ was measured by inversion recovery experiments at 10 mM of TPP, TPPO, TPOP and [Cr(acac)$_3$].*

***Table S11***. *[31]P $T_1$ values of TPP, TPPO and TPOP as measured by inversion recovery experiments from 273 to 353 K under Ar at 10 mM TPP, TPPO and TPOP and 10 mM of [Cr(acac)$_3$] in 0.5 mL of dry non-deuterated toluene.*

| T (K) | $T_1$(TPPO) (s) | $T_1$(TPP) (s) | $T_1$(TPOP) (s) |
|---|---|---|---|
| 273 | 0.36 ± 0.08 | 0.76 ± 0.16 | 0.60 ± 0.10 |
| 278 | 0.47 ± 0.01 | 0.96 ± 0.03 | 0.75 ± 0.02 |
| 283 | 0.52 ± 0.05 | 1.02 ± 0.05 | 0.81 ± 0.06 |
| 288 | 0.54 ± 0.05 | 1.06 ± 0.10 | 0.89 ± 0.05 |
| 293 | 0.48 ± 0.06 | 0.979 ± 0.07 | 0.78 ± 0.08 |
| 298 | 0.68 ± 0.04 | 1.05 ± 0.04 | 1.09 ± 0.05 |
| 303 | 0.74 ± 0.06 | 1.04 ± 0.02 | 1.24 ± 0.08 |
| 308 | 0.76 ± 0.07 | 1.07 ± 0.06 | 1.29 ± 0.10 |
| 313 | 0.88 ± 0.06 | 1.10 ± 0.06 | 1.36 ± 0.11 |
| 318 | 0.89 ± 0.07 | 1.09 ± 0.08 | 1.53 ± 0.06 |
| 323 | 1.07 ± 0.10 | 1.01 ± 0.04 | 1.53 ± 0.06 |
| 328 | 1.00 ± 0.08 | 1.02 ± 0.09 | 1.65 ± 0.12 |
| 333 | 1.10 ± 0.07 | 0.94 ± 0.07 | 1.82 ± 0.08 |
| 338 | 1.15 ± 0.15 | 1.05 ± 0.05 | 1.98 ± 0.07 |
| 343 | 1.15 ± 0.09 | 0.98 ± 0.05 | 1.96 ± 0.11 |
| 348 | 1.14 ± 0.09 | 0.93 ± 0.20 | 2.19 ± 0.13 |
| 353 | 1.13 ± 0.09 | 0.80 ± 0.04 | 2.27 ± 0.14 |

***Table S12***. *[31]P $T_1$ values of TPP, TPPO and TOPO as measured by inversion recovery experiments at different temperatures under Ar in a sample containing 10 mM of TPP, TPPO and TPOP and 10 mM of [Cr(acac)$_3$] in 0.5 mL of dry non-deuterated toluene.*

| T (K) | $T_1$ TPPO (s) | $T_1$ TPP (s) | $T_1$ TPOP (s) |
|---|---|---|---|
| 273 | 0.43 ± 0.03 | 1.10 ± 0.10 | 0.66 ± 0.02 |
| 293 | 0.70 ± 0.07 | 1.44 ± 0.05 | 0.93 ± 0.07 |
| 298 | 0.67 ± 0.05 | 1.60 ± 0.06 | 1.07 ± 0.09 |
| 313 | 0.98 ± 0.07 | 2.07 ± 0.16 | 1.38 ± 0.12 |
| 333 | 1.41 ± 0.12 | 2.49 ± 0.25 | 1.78 ± 0.22 |
| 353 | 1.77 ± 0.15 | 2.51 ± 0.31 | 2.23 ± 0.26 |

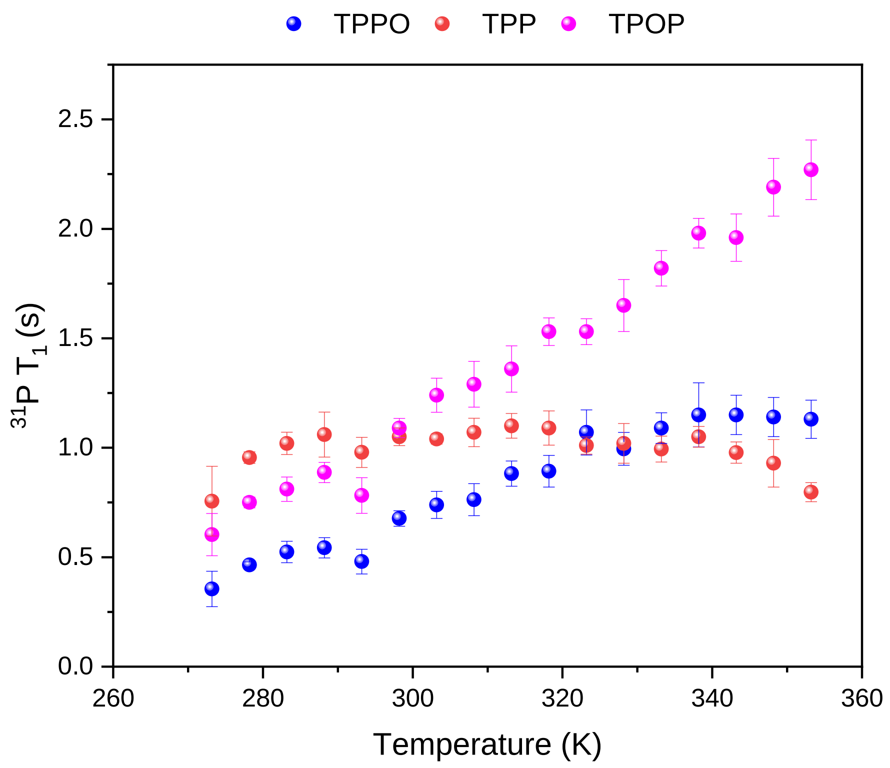


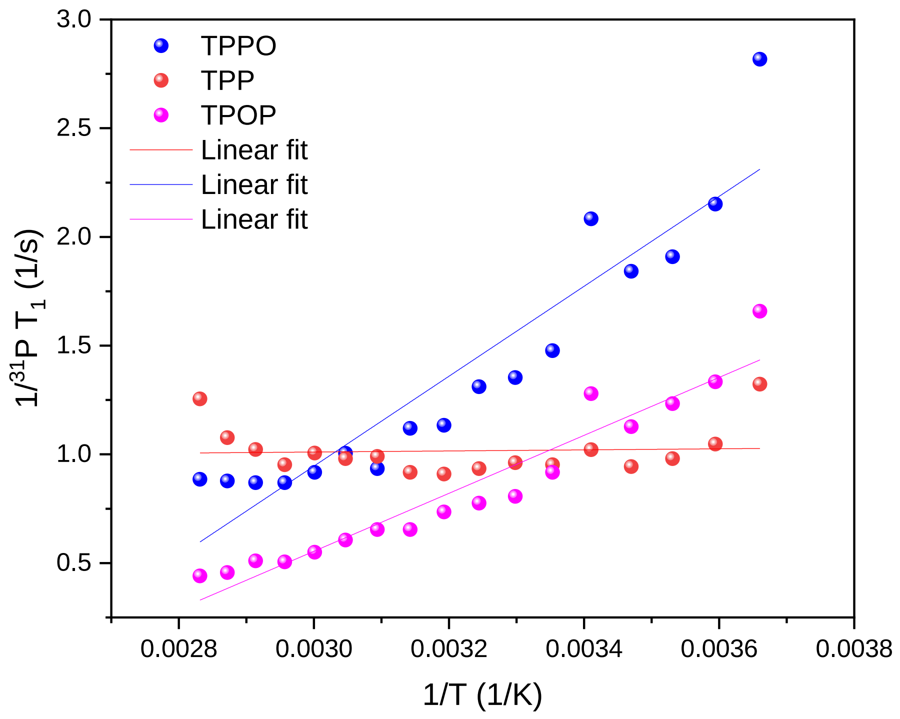


***Figure S6.*** *$^{31}P$ $T_1$ (top) and $1/T_1$ (bottom) values of TPPO (blue), TPP (red) and TPOP (pink) with [Cr(acac)$_3$] at 10 mM each in toluene under Argon at different temperatures.*

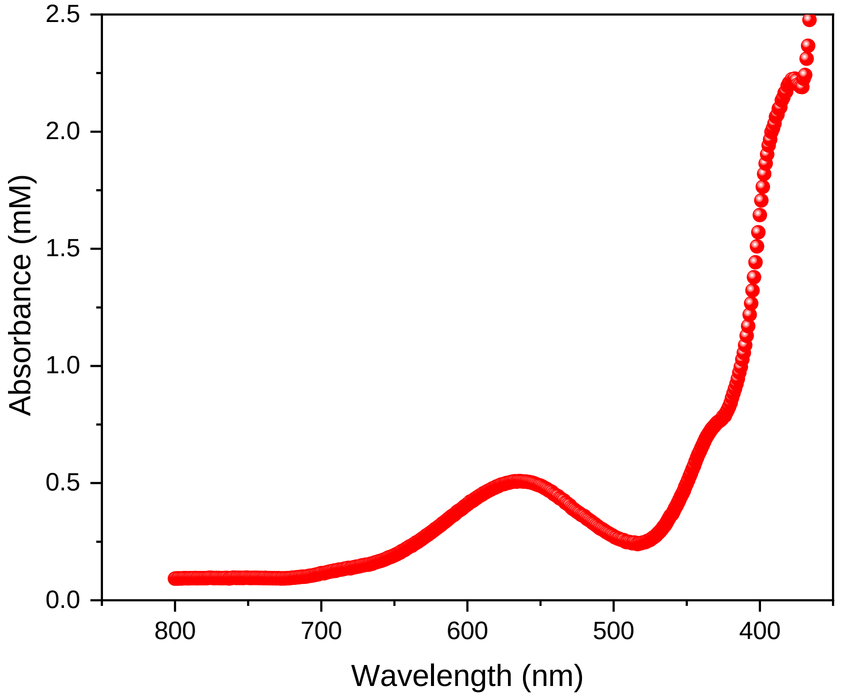


***Figure S7***. *UV-vis spectrum of 10 mM of [Cr(tmhd)$_3$] in non-deuterated toluene.*

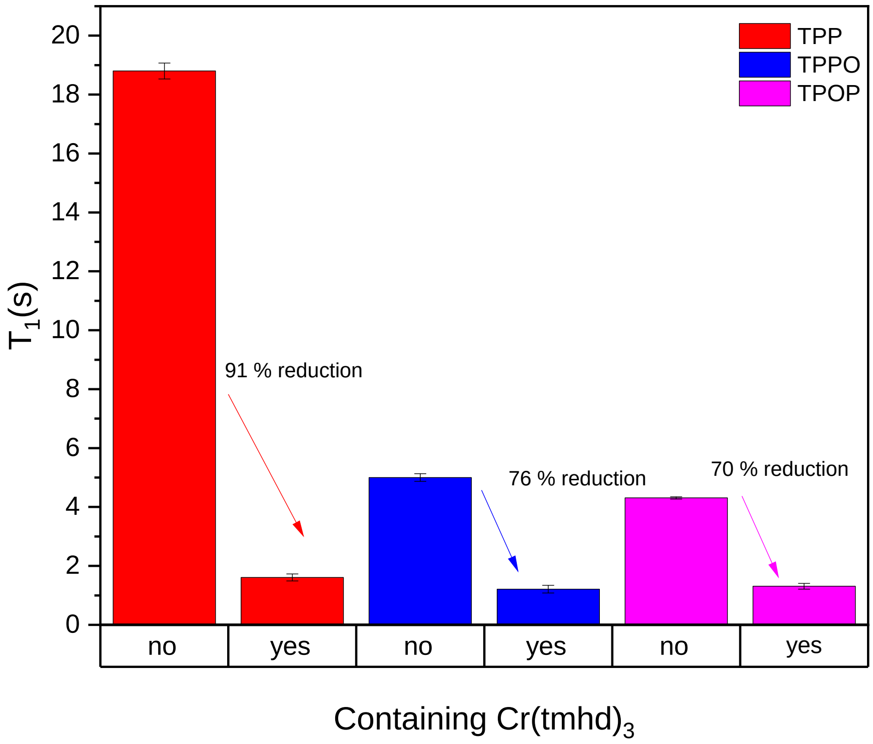


***Figure S8.*** *$^{31}$P $T_1$ values of TPP, TPPO and TPOP as measured by inversion recovery experiments at 298 K under Ar in separate samples containing 10 mM of TPP, TPPO and TPOP and 10 mM of [Cr(tmhd)$_3$] in 0.5 mL of wet non-deuterated toluene.*

***Table S13***. *$^{31}$P $T_1$ values of TPP, TPPO and TPOP as measured by recovery inversion experiments at 298 K under Ar in separate samples containing 10 mM of TPP, TPPO and TPOP and 10 mM of [Cr(tmhd)$_3$] in 0.5 mL of wet non-deuterated toluene.*

| | **TPPO** | **TPP** | **TPOP** |
|---|---|---|---|
| **$T_1$ without PRA (s)** | 5.00 ± 0.13 | 18.80 ± 0.27 | 4.31 ± 0.04 |
| **$T_1$ with [Cr(tmhd)$_3$] (s)** | 1.21 ± 0.13 | 1.61 ± 0.12 | 1.31 ± 0.10 |
| **$T_1$ reduction (%)** | 76 | 91 | 70 |

***Table S14***. *Comparison of chemical shift, peak linewidth and integral of TPP, TPPO and TPOP as recorded by $^{31}$P{$^1$H} spectroscopy at 298 K under Ar after and before heating samples containing 10 mM of each phosphorus species and 10 mM of [Cr(tmhd)$_3$] to 353 K.*

| **Heating** | **Species** | **$\delta$ (ppm)** | **$\Delta\delta$ (ppm)** | **FWHM (Hz)** | **Absolut integral (a.u)** |
|---|---|---|---|---|---|
| **Before** | TPPO | 24.64 | - | 6.58 | 2.55E+7 |
| **After 3 hours** | TPPO | 24.67 | 0.031 | 6.13 | 2.61E+7 |
| **After 3 weeks** | TPPO | 24.79 | 0.15 | 7.34 | 2.57E+7 |
| **Before** | TPP | -4.59 | - | 6.00 | 2.41E+7 |
| **After 3 hours** | TPP | -4.60 | 0.012 | 6.02 | 2.30E+7 |
| **After 3 weeks** | TPP | -4.89 | 0.31 | 6.38 | 2.45E+7 |
| **Before** | TPOP | -16.86 | - | 6.00 | 1.88E+7 |
| **After 3 hours** | TPOP | -16.86 | 0.001 | 6.02 | 1.90E+7 |
| **After 3 weeks** | TPOP | -17.15 | 0.29 | 6.65 | 2.00E+7 |

*__Table S15.__ Comparison of chemical shift, peak linewidth and integral of TPPO and TPP as recorded by $^{1}H$ spectroscopy at 298 K under Ar before and after heating the samples containing 10 mM of each phosphorus species and 10 mM of $[Cr(tmhd)_3]$ to 353 K for three hours.*

| Heating | Species | $\delta$ (ppm) | $\Delta\delta$ (ppm) | FWHM (Hz) | Absolut integral (a.u) |
|---|---|---|---|---|---|
| **Before** | TPPO | 7.65 | - | 23.55 | 5.19E+6 |
| **After** | TPPO | 7.65 | 0.01 | 22.85 | 5.35E+6 |
| **Before** | TPP | 7.29 | - | 12.12 | 4.36E+6 |
| **After** | TPP | 7.28 | 0.01 | 12.36 | 4.36E+7 |

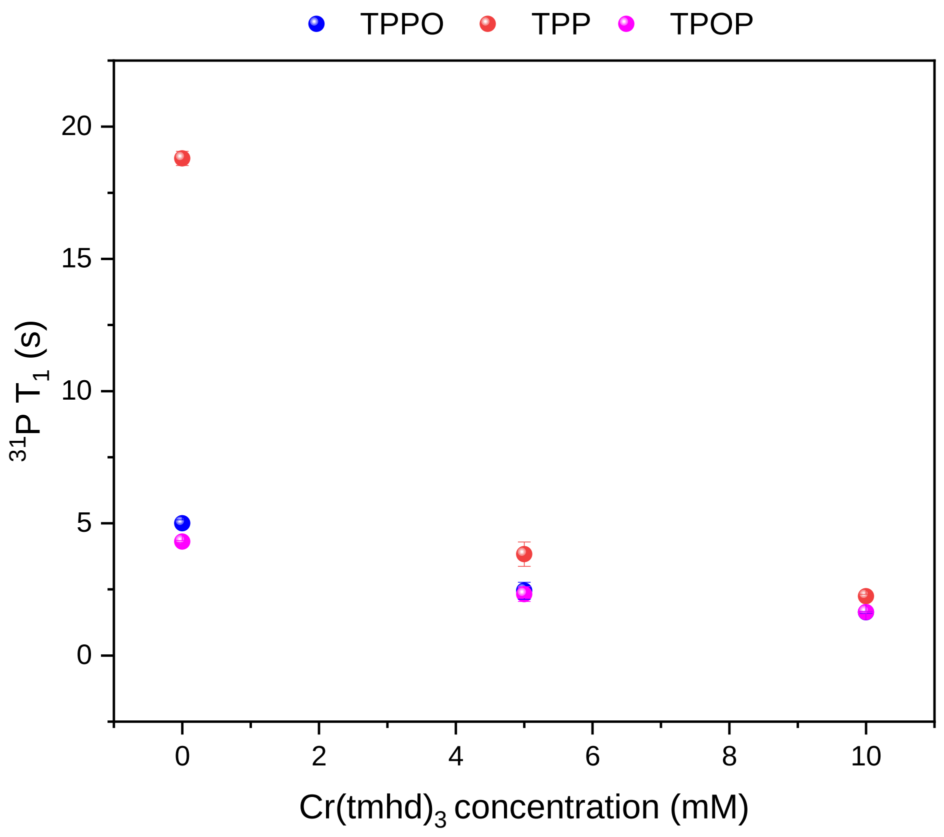


*__Figure S9__. $^{31}P$ $T_1$ values of TPP, TPPO and TPOP as measured by inversion recovery experiments at 298 K under Ar in samples containing 10 mM of TPP, TPPO and TPOP and 0,5 and 10 mM of $[Cr(tmhd)_3]$ in 0.5 mL of dry non-deuterated toluene.*

***Table S16.*** *$^{31}$P $T_1$ values of TPP, TPPO and TPOP as measured by inversion recovery experiments at 298 K under Ar in samples containing 10 mM of TPP, TPPO and TPOP and 5 and 10 mM of [Cr(tmhd)$_3$] in 0.5 mL of wet non-deuterated toluene.*

| [Cr(tmhd)$_3$] (mM) | $T_1$ TPPO (s) | $T_1$ reduction (%) | $T_1$ TPP (s) | $T_1$ reduction (%) | $T_1$ TPOP (s) | $T_1$ reduction (%) |
|---|---|---|---|---|---|---|
| 0 | 4.84 ± 0.12 | - | 17.5 ± 0.58 | - | 4.21 ± 0.12 | - |
| 5 | 2.45 ± 0.32 | 49.38 | 3.83 ± 0.46 | 78.11 | 2.32 ± 0.28 | 44.89 |
| 10 | 1.63 ± 0.03 | 66.32 | 2.24 ± 0.08 | 87.20 | 1.64 ± 0.03 | 61.05 |

***Table S17****. $^{31}$P $T_1$ values of TPP, TPPO and TPOP as measured by inversion recovery experiments at different temperatures under Ar in samples individually containing 10 mM of TPP, TPPO and TPOP and 10 mM of [Cr(tmhd)$_3$] in 0.5 mL of dry non-deuterated toluene.*

| Sample | $T_{set}$ | $T_1$ TPPO (s) | $T_1$ TPP (s) | $T_1$ TPOP (s) |
|---|---|---|---|---|
| **1** | 273 | 0.85 ± 0.04 | - | - |
| | 283 | 1.02 ± 0.07 | - | - |
| | 293 | 1.21 ± 0.08 | - | - |
| | 303 | 1.31 ± 0.14 | - | - |
| | 313 | 1.67 ± 0.05 | - | - |
| | 323 | 1.74 ± 0.17 | - | - |
| | 333 | 1.92 ± 0.28 | - | - |
| | 343 | 2.10 ± 0.24 | - | - |
| | 353 | 2.30 ± 0.13 | - | - |
| **2** | 273 | - | 1.12 ± 0.03 | - |
| | 283 | - | 1.22 ± 0.15 | - |
| | 293 | - | 1.48 ± 0.08 | - |
| | 303 | - | 1.71 ± 0.20 | - |
| | 313 | - | 1.96 ± 0.26 | - |
| | 323 | - | 2.15 ± 0.41 | - |
| | 333 | - | 2.23 ± 0.30 | - |
| | 343 | - | 2.62 ± 0.35 | - |
| | 353 | - | 2.76 ± 0.42 | - |
| **3** | 273 | - | - | 0.91 ± 0.02 |
| | 283 | - | - | 1.07 ± 0.05 |
| | 293 | - | - | 1.25 ± 0.06 |
| | 303 | - | - | 1.51 ± 0.11 |
| | 313 | - | - | 1.59 ± 0.13 |
| | 323 | - | - | 1.65 ± 0.13 |
| | 333 | - | - | 1.81 ± 0.14 |
| | 343 | - | - | 2.07 ± 0.19 |
| | 353 | - | - | 2.16 ± 0.29 |

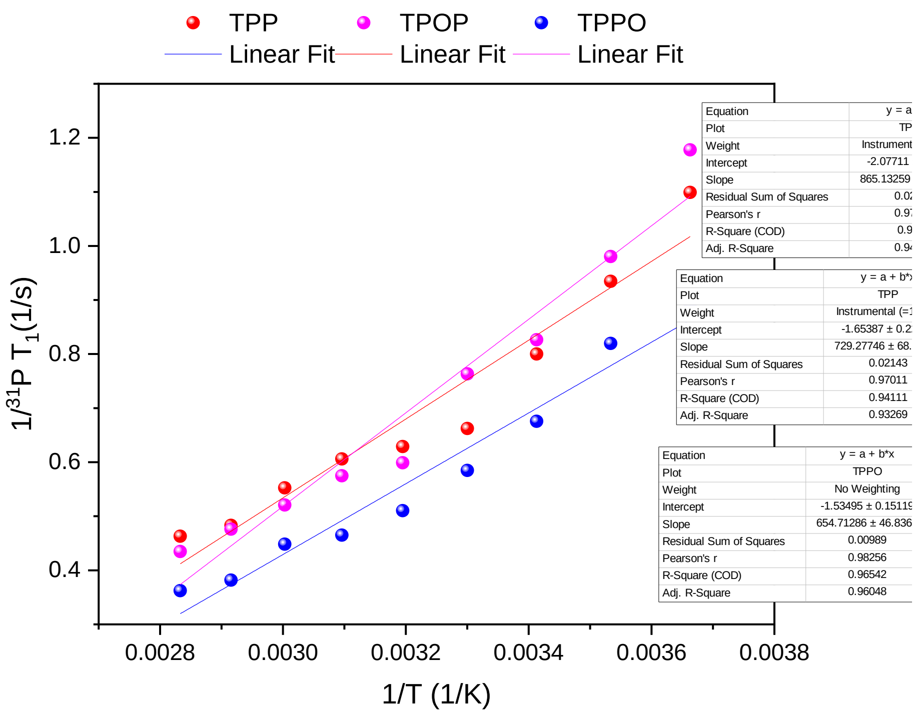


*Figure S10. Linear fits and equations for the 1/T1 of the phosphorus containing species with 10 mM of [Cr(tmhd)$_3$] in dry toluene under Argon at different temperatures.*

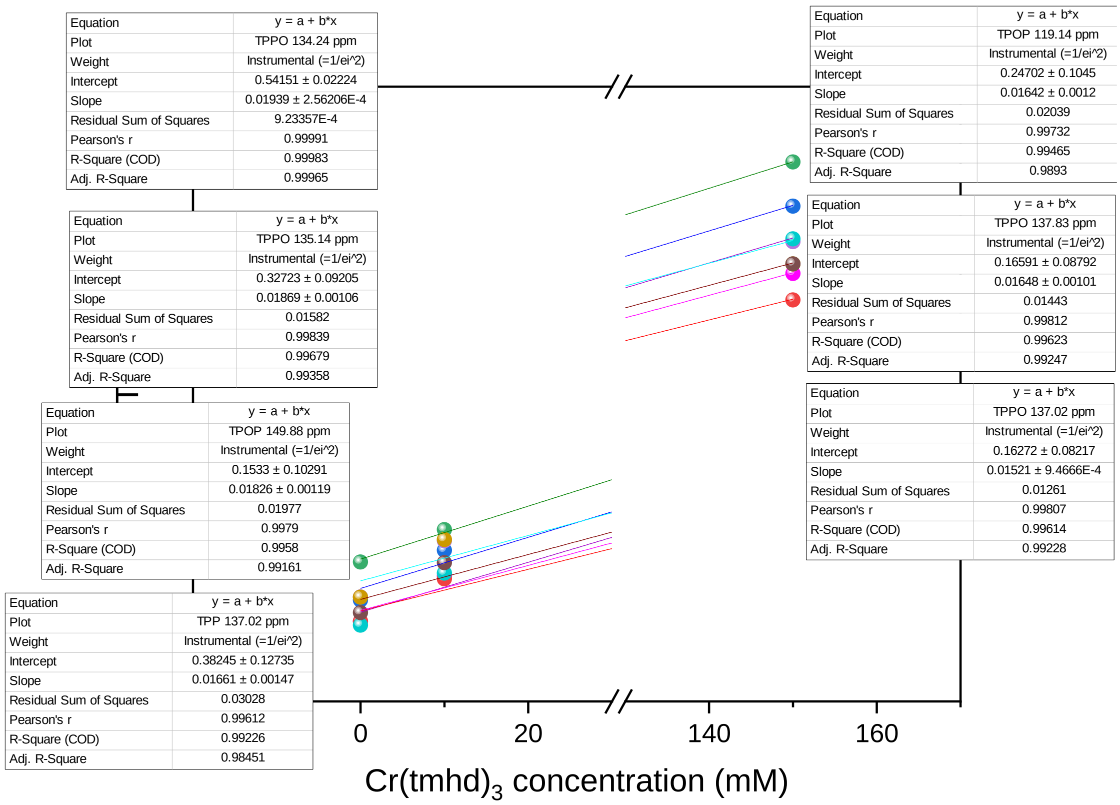


*Figure S11. Linear fits and equations for the 1/T1 of the phosphorus containing species with 0, 10 and 150 mM of [Cr(tmhd)$_3$] in dry toluene under Argon.*

***Table S18.*** *$^{1}H$ $T_1$ values of TPP, TPPO as measured by recovery inversion experiments at 298 K under Ar in individual samples containing 10 mM of TPP, TPPO and TPOP and 10 mM of [Cr(tmhd)$_3$] in 0.5 mL of dry non-deuterated toluene.*

| | $T_1$ TPPO (s) | $T_1$ reduction (%) | $T_1$ TPP (s) | $T_1$ reduction (%) |
|---|---|---|---|---|
| **Without PRA** | 6.25 ± 0.23 | - | 9.88 ± 1.9 | - |
| **With [Cr(tmhd)$_3$]** | 0.31 ± 0.03 | 95.04 | 0.67 ± 0.09 | 93.22 |

***Table S19****. Comparison of chemical shift, peak linewidth and integral of TPPO and TPP as recorded by $^{1}H$ NMR spectroscopy at 298 K under Ar in individual samples containing 10 mM of each phosphorus species in the presence/absence of 10 mM of [Cr(tmhd)$_3$].*

| [Cr(tmhd)$_3$] | Species | $\delta$ (ppm) | $\Delta\delta$ (ppm) | FWHM (Hz) | Absolut integral (a.u) |
|---|---|---|---|---|---|
| No | TPPO | 7.65 | - | 22.40 | 5.19E+6 |
| Yes | TPPO | 7.65 | 0.01 | 22.84 | 5.20E+6 |
| No | TPP | 7.29 | - | 11.08 | 4.24E+6 |
| Yes | TPP | 7.28 | 0.01 | 12.12 | 4.36E+7 |

***Table S20****. $^{13}C$ $T_1$ values of TPPO as measured by inversion recovery experiments at 298 K under Ar in a sample containing 150 mM of TPPO and 10 or 150 mM of [Cr(tmhd)$_3$] in 0.5 mL of dry non-deuterated toluene.*

| [Cr(tmhd)$_3$] (mM) | $T_1$ TPPO (s) at 137.83 ppm | $T_1$ reduction (%) | $T_1$ TPPO (s) at 137.02 ppm | $T_1$ reduction (s) | $T_1$ TPPO (s) at 135.14 ppm | $T_1$ reduction (%) | $T_1$ TPPO (s) at 134.24 ppm | $T_1$ reduction (%) |
|---|---|---|---|---|---|---|---|---|
| 0 | 11.90 ± 2.78 | - | 11.60 ± 2.17 | - | 4.14 ± 0.06 | - | 1.92 ± 0.15 | - |
| 10 | 2.39 ± 0.66 | 79.92 | 2.52 ± 0.56 | 78.28 | 1.65 ± 0.32 | 60.14 | 1.32 ± 0.24 | 31.25 |
| 150 | 0.38 ± 0.16 | 96.81 | 0.41 ± 0.07 | 96.47 | 0.32 ± 0.06 | 92.27 | 0.29 ± 0.06 | 84.90 |

***Table S21****. $^{13}C$ $T_1$ values of TPP as measured by inversion recovery experiments at 298 K under Ar in samples containing 150 mM of TPP and 10 or 150 mM of [Cr(tmhd)$_3$] in 0.5 mL of dry non-deuterated toluene.*

| [Cr(tmhd)$_3$] (mM) | $T_1$ TPP (s) at 137.02 ppm | $T_1$ reduction (%) | $T_1$ TPP (s) at 136.81 ppm | $T_1$ reduction (%) |
|---|---|---|---|---|
| 0 | 3.79 ± 0.85 | - | 3.80 ± 0.86 | - |
| 10 | 1.48 ± 0.21 | 60.94 | 1.46 ± 0.20 | 61.58 |
| 150 | 0.349 ± 0.33 | 90.80 | 0.37 ± 0.34 | 90.26 |

***Table S22.*** *$^{13}$C $T_1$ values of TPOP as measured by inversion recovery experiments at 298 K under Ar in samples containing 150 mM of TPOP and 10 or 150 mM of [Cr(tmhd)$_3$] in 0.5 mL of dry non-deuterated toluene.*

| [Cr(tmhd)$_3$] (mM) | $T_1$ TPOP (s) at 149.88 ppm | $T_1$ reduction (%) | $T_1$ TPOP (s) at 119.14 ppm | $T_1$ reduction (%) |
|---|---|---|---|---|
| 0 | 17.4 ± 2.24 | - | 6.68 ± 0.60 | - |
| 10 | 2.28 ± 0.65 | 86.90 | 1.94 ± 0.27 | 70.96 |
| 150 | 0.35 ± 0.12 | 97.99 | 0.37 ± 0.05 | 94.46 |

***Table S23.*** *Comparison of chemical shift, peak linewidth and integral of TTPO, TPP and TPOP cross-peaks as recorded by $^{1}$H-$^{13}$C HMBC NMR spectroscopy at 298 K under Ar in individual samples containing 150 mM of each phosphorus species in the presence/absence of 10 mM of [Cr(tmhd)$_3$] .*

| [Cr(tmhd)$_3$] | Species | $\delta$ (ppm) (f2, f1) | FWHM (Hz) (f2, f1) | Absolut integral (a.u) |
|---|---|---|---|---|
| No | TPPO | 6.64,22.65 | 38.92 ,238.12 | 1.39E+8 |
| Yes | TPPO | 8.28,24.41 | 37.99, 239.12 | 4.53E+8 |
| No | TPP | 6.20, -6.17 | 33.06, 239.12 | 5.61E+8 |
| Yes | TPP | 7.96, -5.00 | 30.24, 240.33 | 2.33E+9 |
| No | TPOP | 7.44, -16.76 | 32.36, 239.01 | 2.54E+7 |
| Yes | TPOP | 7.83, -16.76 | 32.52, 239.12 | 6.62E+7 |

***Table S24.*** *$^{31}$P $T_1$ values of **D**, **F** and **G** as measured by inversion recovery experiments at 298 K under Ar in individual samples containing 10 mM of **D**, **F** and **G** and 10 mM of [Cr(tmhd)$_3$] in 0.5 mL of dry non-deuterated toluene.*

| | $T_1$ (D) (s) | $T_1$ (F) (s) | $T_1$ (G) (s) at 41.84 ppm |
|---|---|---|---|
| **without [Cr(tmhd)$_3$]** | 1.54 ± 0.23 | 0.78 ± 0.10 | 1.97 ± 0.76 |
| **with [Cr(tmhd)$_3$]** | 1.02 ± 0.19 | 0.50 ± 0.07 | 1.28 ± 0.60 |
| **reduction (%)** | 34 | 36 | 35 |

***Table S25.*** *$^{31}$P and $^{1}$H $T_1$ values of **E** as measured by inversion recovery experiments at 298 K under Ar in individual samples containing 10 mM of **E** and 10 mM of [Cr(tmhd)$_3$] in 0.5 mL of dry non-deuterated toluene.*

| | $^{31}$P $T_1$ (E) (s) | $^{1}$H $T_1$ (E) (s) |
|---|---|---|
| **without [Cr(tmhd)$_3$]** | 1.38 ± 0.36 | 0.73 ± 0.11 |
| **with [Cr(tmhd)$_3$]** | 0.82 ± 0.12 | 0.27 ± 0.07 |
| **reduction (%)** | 41 | 63 |

***Table S26.*** *Comparison of chemical shift, peak linewidth and integral of **D**, **E**, **F** and **G** as recorded by $^{1}H$ and $^{31}P\{^{1}H\}$ NMR spectroscopy at 298 K under Ar in individual samples containing 10 mM of each complex in the presence/absence of 10 mM of [Cr(tmhd)$_3$].*

| **[Cr(tmhd)$_3$]** | **Complex** | **$\delta$ (ppm)** | **$\Delta\delta$ (ppm)** | **FWHM (Hz)** | **Absolut integral (a.u)** |
|---|---|---|---|---|---|
| No | **D** | 10.71 | - | 13.31 | 1.71E+06 |
| Yes | **D** | 11.22 | -0.51 | 15.19 | 1.71E+06 |
| No | **D** | -141.86 | | 1434.59 | 1.15E+06 |
| Yes | **D** | -144.28 | 2.42 | 1437.08 | 1.27E+06 |
| No | **E** | 40.53 | - | 187.21 | 2.00E+07 |
| Yes | **E** | 40.73 | 0.20 | 186.47 | 2.35E+07 |
| No | **E** | -9.31 | - | 22.89 | 1.40E+07 |
| Yes | **E** | -9.32 | 0.01 | 21.35 | 1.46E+07 |
| No | **F** | 85.05 | - | 7.28 | 1.79E+07 |
| Yes | **F** | 85.39 | 0.34 | 7.68 | 1.88E+07 |
| No | **G** | 43.00 | - | 15.92 | 9.25E+06 |
| Yes | **G** | 42.30 | -0.80 | 12.61 | 9.31E+06 |
| No | **G** | -16.64 | - | 13.81 | 5.07E+07 |
| Yes | **G** | -17.66 | -1.02 | 11.51 | 5. 36E+07 |

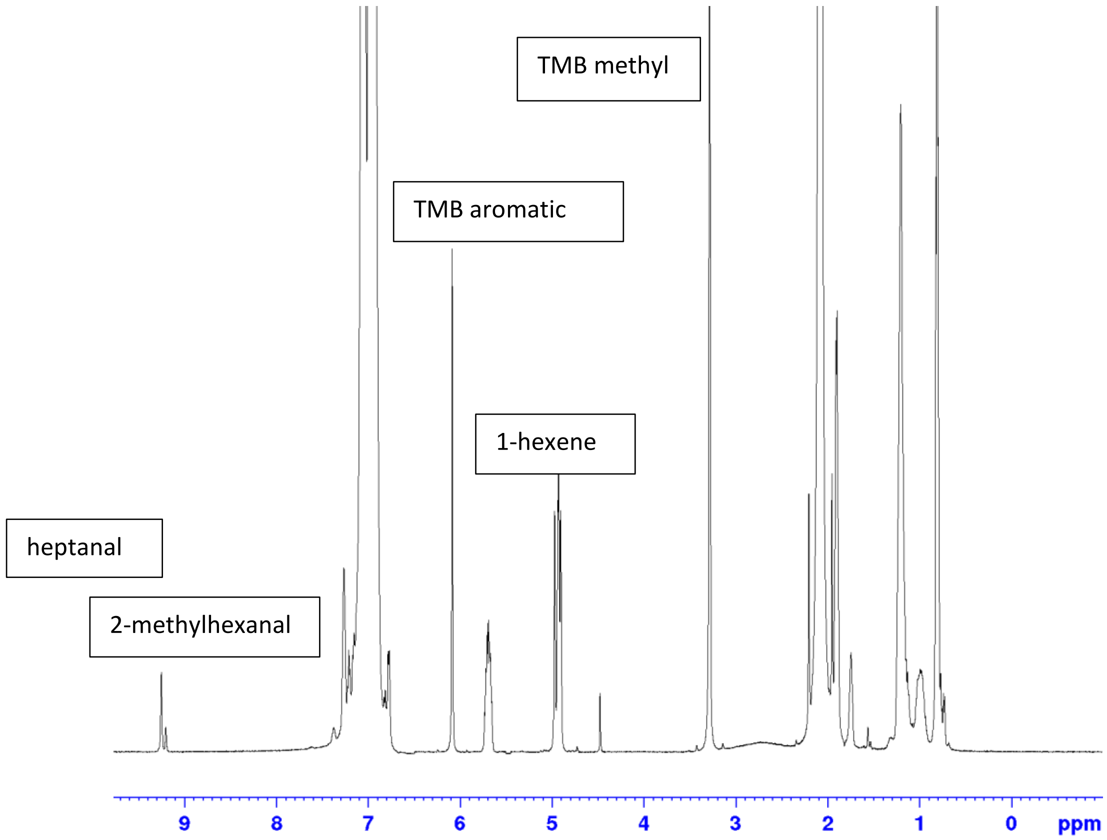


***Figure S12.*** *$^{1}$H NMR spectrum recorded at flow conditions during the hydroformylation of 1-hexene.*

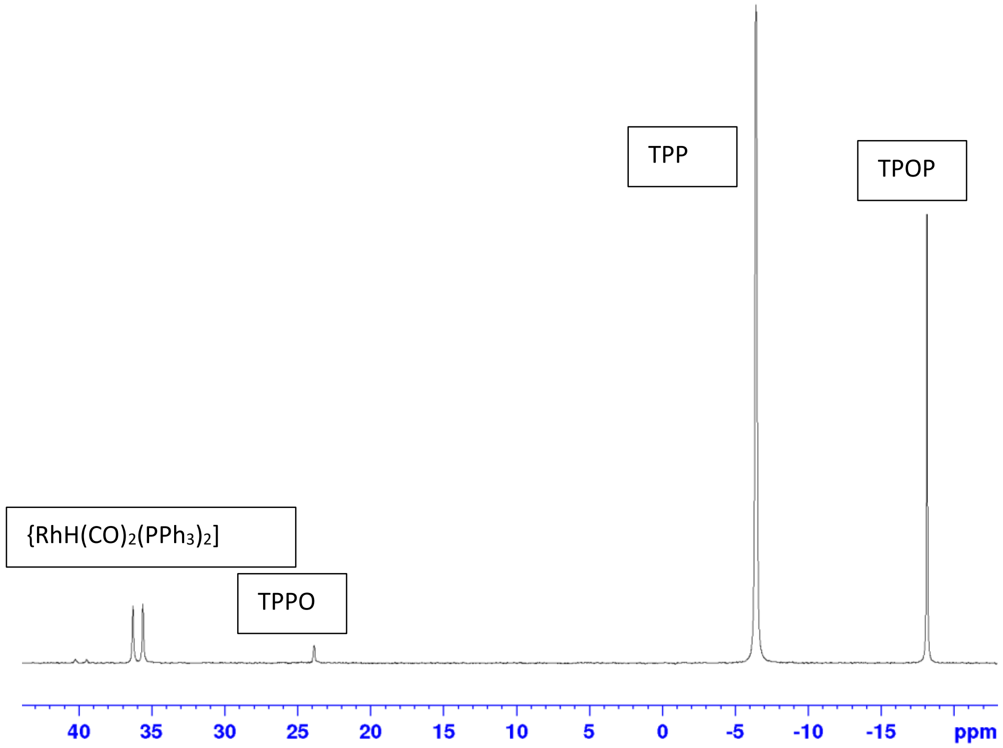


***Figure S13***. *$^{31}$P{$^{1}$H} NMR spectrum recorded at flow conditions during the hydroformylation of 1-hexene*

***Table S27.*** Signal-to-noise *(S/N) and CF of the most important resonances in the reaction mixture as recorded by $^{1}H$ or $^{31}P\{^{1}H\}$ FlowNMR spectroscopy at 0 ℃ during the hydroformylation of 1-hexene catalysed by [Rh(acac)(CO)$_2$] = 2.5 mM and TPP = 50 mM under 10 bar of $H_2$/CO (1:1) at 50 ℃ (without PRA).*

| | 1-hexene | 2-methylhexanal | heptanal | TMB aromatic | TMB methyl | TPPO | TPP | TPOP | [RhH(CO)$_2$(PPh$_3$)$_2$] ($^{1}H/^{31}P$) |
|---|---|---|---|---|---|---|---|---|---|
| **S/N** | 1080.1 | 100.3 | 292.6 | 1068.5 | 2928.4 | 15.5 | 355.7 | 741.74 | 602.7/73.7 |
| **CF** | 4.29 | 4.82 | 4.93 | 2.74 | 1.72 | 5.64 | 11.62 | 4.94 | 1.23/2.88 |

***Table S28****. $^{1}H$ $T_1$ values of heptanal, 2-methylhexanal, 1-hexene and 1,3,5-trimethoxybenzene (TMB) as measured at static conditions by inversion recovery experiments at 0 °C under 10 bar of $H_2$/CO(1:1) during the hydroformylation reaction of 1-hexene catalysed PPh$_3$/[Rh(acac)(CO)$_2$]. [Rh(acac)(CO)$_2$] = 2.5 mM and TPP = 50 Mm (without PRA)*

| $^{1}H$ $T_1$(heptanal) (s) | $^{1}H$ $T_1$(2-methylhexanal) (s) | $^{1}H$ $T_1$(TMB aromatic) (s) | $^{1}H$ $T_1$(1-hexene) (s) | $^{1}H$ $T_1$(TMB methyl) (s) |
|---|---|---|---|---|
| 8.65 ± 0.18 | 8.55 ± 0.13 | 5.59 ± 0.055 | 8.01± 0.047 | 3.06 ± 0.012 |

***Table S29.*** *CF of the most important resonances in the solution as recorded by $^{1}H$ or $^{31}P\{^{1}H\}$ FlowNMR spectroscopy at 0 °C during the hydroformylation of 1-hexene catalysed by [Rh(acac)(CO)$_2$] = 2.5 mM and TPP = 50 mM under 10 bar of $H_2$/CO (1:1) containing 10 mM of [Cr(tmhd)$_3$] (with PRA).*

| | 1-hexene | 2-methylhexanal | heptanal | TMB aromatic | TMB methyl | TPPO | TPP | TPOP | [RhH(CO)$_2$(PPh$_3$)$_2$] ($^{1}H/^{31}P$) |
|---|---|---|---|---|---|---|---|---|---|
| **CF** | 0.96 | 0.95 | 1.00 | 0.98 | 0.98 | 2.33 | 2.76 | 1.87 | 1.01/1.74 |

***Table S30****. $^{1}H$ $T_1$ values of heptanal, 2-methylhexanal and 1,3,5-trimethoxybenzene (TMB) as measured by inversion recovery experiments at 0 °C under 10 bar of $H_2$/CO during the hydroformylation reaction of 1-hexene catalysed PPh$_3$/[Rh(acac)(CO)$_2$]. [Rh(acac)(CO)$_2$] = 2.5 mM and TPP = 50 mM containing 10 mM of [Cr(tmhd)$_3$] (with PRA). % reduction calculated compared with the values obtained in the absence of PRA.*

| | $^{1}H$ $T_1$(heptanal) (s) | $^{1}H$ $T_1$(2-methylhexanal) (s) | $^{1}H$ $T_1$(TMB aromatic) (s) | $^{1}H$ $T_1$(1-hexene) (s) | $^{1}H$ $T_1$(TMB methyl) (s) |
|---|---|---|---|---|---|
| **$T_1$** | 0.50 ± 0.007 | 0.63 ± 0.006 | 0.41 ± 0.037 | 0.41 ± 0.15 | 0.30 ± 0.012 |
| **$T_1$ reduction (%)** | 94.2 % | 92.6 % | 92.7 % | 94.9 % | 90.2 % |

***Table S31***. *$^{31}$P $T_1$ values of [Rh(CO)$_2$(PPh$_3$)$_2$], TPPO, TPOP and TPP as measured by inversion recovery experiments at 0 °C under 10 bar of $H_2$/CO during the hydroformylation reaction of 1-hexene catalysed PPh$_3$/[Rh(acac)(CO)$_2$]. [Rh(acac)(CO)$_2$] = 2.5 mM and TPP = 50 mM containing 10 mM of [Cr(tmhd)$_3$] (with PRA). % reduction calculated compared with the values obtained in the absence of PRA.*

| | $^{31}$P $T_1$(RhH(CO)$_2$(PPh$_3$)$_2$) (s) | $^{31}$P $T_1$(TPPO) (s) | $^{31}$P $T_1$(TPP) (s) | $^{31}$P $T_1$(TPOP) (s) |
|---|---|---|---|---|
| $T_1$ | 1.02 ± 0.11 | 1.51 ± 0.15 | 1.78 ± 0.09 | 1.29 ± 0.17 |
| $T_1$ reduction (%) | - | 69.8 % | 90.5 % | 70.1 % |

***Table S32.*** Signal-to-noise *(S/N) and CF of the most important resonances in the solution as recorded by $^1$H or $^{31}$P{$^1$H} FlowNMR spectroscopy at 0 °C during the hydroformylation of 1-hexene catalysed by [Rh(acac)(CO)$_2$] = 2.5 mM and TPP = 50 mM under 10 bar of $H_2$/CO (1:1) and containing 10 mM of [Cr(tmhd)$_3$] (with PRA after optimisation of NMR parameters).*

| | 1-hexene | 2-methylhexanal | heptanal | TMB aromatic | TMB methyl | TPPO | TPP | TPOP | [RhH(CO)$_2$(PPh$_3$)$_2$] ($^1$H/$^{31}$P) |
|---|---|---|---|---|---|---|---|---|---|
| S/N | 455.54 | 239.51 | 803.5 | 1265.93 | 2813.62 | 71.42 | 1223.16 | 818.7 | 710.59/91.31 |
| CF | 1.06 | 1.05 | 1.04 | 1.06 | 1.72 | 2.41 | 2.95 | 1.98 | 1.02/2.06 |

***Table S33***. *$^1$H $T_1$ values of heptanal, 2-methylhexanal, 1-hexene and 1,3,5-trimethoxybenzene (TMB) as measured by inversion recovery experiments 0 °C under 10 bar of $H_2$/CO during the hydroformylation reaction of 1-hexene catalysed PPh$_3$/[Rh(acac)(CO)$_2$]. [Rh(acac)(CO)$_2$] = 2.5 mM and TPP = 50 mM containing 10 mM of [Cr(tmhd)$_3$] (with PRA after optimisation of NMR parameters).*

| $^1$H $T_1$(heptanal) (s) | $^1$H $T_1$(2-methylhexanal) (s) | $^1$H $T_1$(TMB aromatic) (s) | $^1$H $T_1$(1-hexene) (s) | $^1$H $T_1$(TMB methyl) (s) |
|---|---|---|---|---|
| 0.58 ± 0.007 | 0.63 ± 0.023 | 0.56 ± 0.084 | 0.63 ± 0.057 | 0.37 ± 0.002 |

***Table S34.*** *$^1$H $T_1$ values of heptanal, 2-methylhexanal, and 1,3,5-trimethoxybenzene (TMB) as measured by inversion recovery experiments 0 °C under Ar during the hydroformylation reaction of 1-hexene catalysed PPh$_3$/[Rh(acac)(CO)$_2$]. [Rh(acac)(CO)$_2$] = 2.5 mM and TPP = 50 mM containing 10 mM of [Cr(tmhd)$_3$] (with PRA after optimisation of NMR parameters).*

| $^1$H $T_1$(heptanal) (s) | $^1$H $T_1$(2-methylhexanal) (s) | $^1$H $T_1$(TMB aromatic) (s) | $^1$H $T_1$(TMB methyl) (s) |
|---|---|---|---|
| 0.50 ± 0.059 | 0.46 ± 0.091 | 0.11 ± 0.074 | 0.26 ± 0.042 |

***Table S35***. *$^{31}$P $T_1$ values [RhH(CO)$_2$(PPh$_3$)$_2$] , TPPO, TPP and TPOP as measured by inversion recovery experiments at 0 °C under Ar during the hydroformylation reaction of 1-hexene catalysed PPh$_3$/[Rh(acac)(CO)$_2$]. [Rh(acac)(CO)$_2$] = 2.5 mM and TPP = 50 mM containing 10 mM of [Cr(tmhd)$_3$] (with PRA after optimisation of NMR parameters).*

| $^{31}$P $T_1$(RhH(CO)$_2$(PPh$_3$)$_2$) (s) | $^{31}$P $T_1$(TPPO) (s) | $^{31}$P $T_1$(TPP) (s) | $^{31}$P $T_1$(TPOP) (s) |
|---|---|---|---|
| 1.87 ± 0.43 | 1.90 ± 0.34 | 1.58 ± 0.10 | 1.51 ± 0.096 |

***Table S36***. *CFs of the most important resonances in the solution as recorded by $^{13}C\{^1H\}$ FlowNMR spectroscopy at 0 °C during the hydroformylation of 1-hexene catalysed by [Rh(acac)(CO)$_2$] = 2.5 mM and TPP = 7.5 mM under 10 bar of $H_2$/CO (1:1).*

| | **TMB** | **2-methylhexanal** | **heptanal** | **internal 1-hexene** | **terminal 1-hexene** |
|---|---|---|---|---|---|
| CF | 14.25 | 11.80 | 9.18 | 9.74 | 9.18 |

## 6. References


1 A. M. R. Hall, J. C. Chouler, A. Codina, P. T. Gierth, J. P. Lowe and U. Hintermair, *Catal. Sci. Technol.*, 2016, **6**, 8406–8417.

2 M. H. Levitt, *Spin dynamics: basics of nuclear magnetic resonance*, John Wiley & Sons, 2013.
